# High-throughput CRISPR technology: a novel horizon for solid organ transplantation

**DOI:** 10.3389/fimmu.2023.1295523

**Published:** 2024-01-04

**Authors:** Xiaohan Li, Zhang Chen, Weicong Ye, Jizhang Yu, Xi Zhang, Yuan Li, Yuqing Niu, Shuan Ran, Song Wang, Zilong Luo, Jiulu Zhao, Yanglin Hao, Junjie Zong, Chengkun Xia, Jiahong Xia, Jie Wu

**Affiliations:** ^1^ Department of Cardiovascular Surgery, Union Hospital, Tongji Medical College, Huazhong University of Science and Technology, Wuhan, China; ^2^ Center for Translational Medicine, Union Hospital, Tongji Medical College, Huazhong University of Science and Technology, Wuhan, China; ^3^ Department of Anesthesiology, Union Hospital, Tongji Medical College, Huazhong University of Science and Technology, Wuhan, China; ^4^ Key Laboratory of Organ Transplantation, Ministry of Education, National Health Commission (NHC) Key Laboratory of Organ Transplantation, Key Laboratory of Organ Transplantation, Chinese Academy of Medical Sciences, Wuhan, China

**Keywords:** solid organ transplantation, CRISPR/Cas9, transcriptome editing, CRISPR screening, high-throughput

## Abstract

Organ transplantation is the gold standard therapy for end-stage organ failure. However, the shortage of available grafts and long-term graft dysfunction remain the primary barriers to organ transplantation. Exploring approaches to solve these issues is urgent, and CRISPR/Cas9-based transcriptome editing provides one potential solution. Furthermore, combining CRISPR/Cas9-based gene editing with an *ex vivo* organ perfusion system would enable pre-implantation transcriptome editing of grafts. How to determine effective intervention targets becomes a new problem. Fortunately, the advent of high-throughput CRISPR screening has dramatically accelerated the effective targets. This review summarizes the current advancements, utilization, and workflow of CRISPR screening in various immune and non-immune cells. It also discusses the ongoing applications of CRISPR/Cas-based gene editing in transplantation and the prospective applications of CRISPR screening in solid organ transplantation.

## 1 Introduction

The CRISPR/Cas9 system is a third-generation genome editing technology that has emerged as an advanced replacement for zinc-finger nucleases and transcription activator-like effector nucleases in gene editing due to its improved simplicity and precision (1). CRISPR/Cas systems and their derivatives can be used for single genetic procedures and high-throughput studies, including pooled and arrayed screening (2). Feng et al. (3) and Langer and Sabatini (4) introduced the first high-throughput CRISPR screening in 2014. CRISPR screening has enabled comprehensive transcriptome analyses and improved mechanistic insights into gene regulation networks (5). Over the past decades, numerous genome-scale RNA interference (RNAi) screens have been conducted in biomedical studies. Unlike CRISPR-based technologies, it does not require co-delivery of exogenous proteins (6). However, due to off-target effects as well as differences in knockdown efficiencies, RNA interference (RNAi) screens exhibit limited validation or overlap across studies (6). Fortunately, CRISPR screenings demonstrate more pronounced phenotypic effects, higher validation rates, greater result consistency, reproducible data, and minimal off-target effects (7, 8). CRISPR screening has been used in multiple immunological fields to explore potential targets in drug therapy or cellular immunotherapy, including cancer, infection, and autoimmune disease (9–11). However, CRISPR screening has not been used in the organ transplantation field.

The success rate of organ transplantation has significantly increased in recent years due to advances in surgical techniques, perioperative care, immunosuppressive drugs, and infectious disease management. Organ transplantation has become the primary therapeutic approach for patients with terminal organ failure. However, the lack of available grafts and poor long-term prognosis remain significant challenges to organ transplantation. Therefore, there is an urgent need for alternative approaches to increase both the quantity and longevity of grafts in transplantation. CRISPR/Cas9-mediated transcriptome editing is one potential solution. It provides a promising approach to precisely alter potential intervention targets in multiple cells. Moreover, combining the CRISPR/Cas9 technique with an *ex vivo* organ perfusion system enables pre-implantation transcriptome editing of grafts (12, 13). How to select effective targets becomes the new challenge. Fortunately, CRISPR screening has facilitated the identification of potential therapeutic targets within immune and non-immune cells in various immune-related diseases (5), offering a novel insight for transplantation.

This review provides a comprehensive overview of the current advancements and utilization of CRISPR screenings in diverse immune and non-immune cells, providing valuable guidance for applying these techniques in organ transplantation research. Then, we summarize the ongoing applications of CRISPR/Cas-based gene editing in transplantation and the prospective applications of CRISPR screening in solid organ transplantation.

## 2 Current application of high-throughput CRISPR technology in solid organ transplantation

The long-term graft dysfunction mediated by allogeneic rejection remain the primary barriers to organ transplantation. Numerous immunosuppressants have been developed for transplantation, including calcineurin inhibitors and mTOR inhibitors (14, 15). However, most of these have limited therapeutic effects on long-term allograft survival (16). In addition, cellular immunomodulatory therapies have been proven effective in suppressing the immune response in transplantation (17). Regulatory T cells (Tregs) are currently employed to suppress immune responses and promote antigens tolerance in clinical (18). Additionally, multiple suppressor cells, including regulatory macrophages, tolerogenic dendritic cells (DCtols), and mesenchymal stromal cells (MSCs), are under investigation in clinical trials (16). Moreover, chimeric antigen receptor (CAR) T-cell therapy has emerged as a potent therapeutic approach to achieve long-term graft survival. Recent studies have shown that CAR technology can be applied successfully to create Treg cells based on donor HLA molecules, and can overcome the limitations of existing protocols for enriching Tregs based on repetitive stimulation with alloantigen (19). Unfortunately, these therapeutic cells usually cannot maintain the intended function to effectively build the tolerance in solid organ transplantation (16). Enhancing the effector function of therapeutic cells with CRISPR/Cas9 based genome editing, providing one potential solution (20–22). However, current studies have identified limited genetic targets that can effectively regulate the immune cells in transplantation (23). Exploring new intervention targets for immunosuppressant and cellular immunomodulatory therapies is urgent. Furthermore, combining CRISPR/Cas9-based gene editing with *ex vivo* organ perfusion system would enable pre-implantation transcriptome editing of grafts (12). This is a potential way to solve the shortage of available grafts. However, how to select resistance genes becomes the new challenge.

Fortunately, high-throughput CRISPR screening has dramatically accelerated the effective targets in various immunocytes (2). However, its application in solid organ transplantation is limited. Therefore, we have provided an overview of the current utilization of CRISPR screenings in diverse immune cells, providing insights for screening therapeutic targets of immunocytes in solid organ transplantation. Moreover, CRISPR screens had emerged as a powerful way to explore the potential targets in immune escape mechanism of cancer cells under immune pressure (24). In solid organ transplantation, grafts face a similar immune pressure, specifically mediated by allograft rejection. This provides a novel insight for exploring the resistance genes in solid organ transplantation. A comprehensive discussion of CRISPR screenings in cancer cells is also presented in the subsequent sections, providing guidance for conducting CRISPR screenings in grafts.

## 3 CRISPR/Cas9 system: a reliable and effective genome editing tool

Precise genetic manipulation techniques in living cells have significantly contributed to advancements in biomedical research and the application of genetic perturbation in clinical medicine (25). Protein-DNA recognition is a critical aspect of genome editing and plays a pivotal role in the precise interaction between genetic editing enzymes and DNA targets (26). However, site-specific nucleic acid targeting is a significant challenge in genome editing strategies based on protein-DNA recognition (27). Numerous studies have elucidated the protein-DNA recognition mechanism (26, 28–30). One of the most significant ongoing discussions in the specific genetic editing field is the CRISPR/Cas system (31–33).

**Figure 1:**
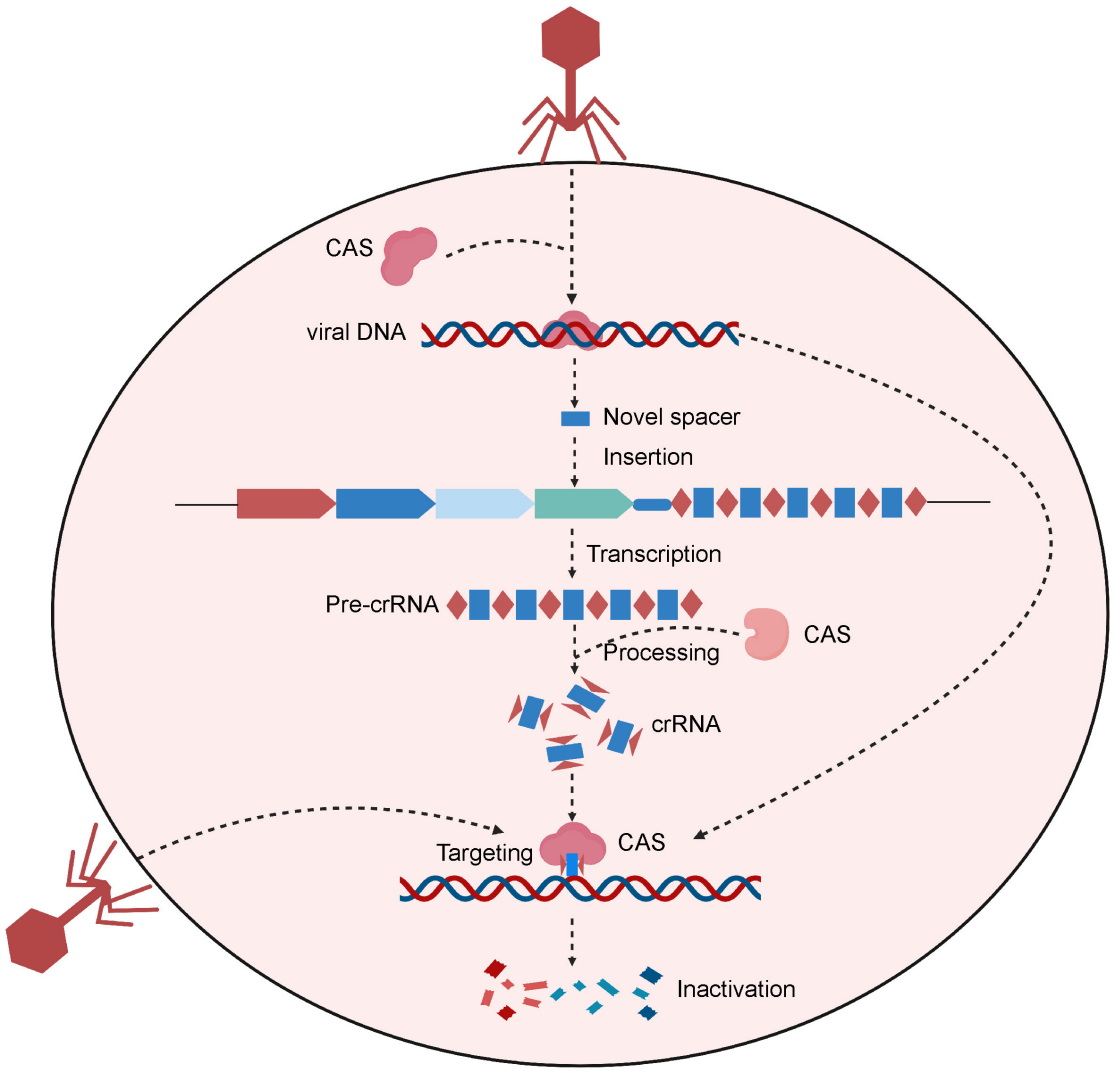
Schematic of the CRISPR-Cas system of the natural prokaryotic immune system. After viral invasion, certain bacteria can incorporate a viral gene fragment into their CRISPR storage space. When reencountering the virus, bacteria initiate CRISPR transcription to produce pre-crRNA, which are then processed to create crRNAs. Upon recognizing sequences homologous to the viral gene, the crRNAs guide Cas protein to bind to and cleave the target gene, protecting the bacterial host from viral infiltration. Created with BioRender.com.

The CRISPR/Cas system is a type of natural immune system in prokaryotes (34). CRISPR stands for “Clustered Regularly Interspaced Short Palindromic Repeats”. Prokaryotes use this mechanism to counteract invading viruses and foreign DNA (35) (**Figure 1**). Upon viral invasion, certain bacteria can incorporate a viral gene fragment into their DNA within a designated region, known as the CRISPR storage space (36). When reencountering the virus, bacteria initiate CRISPR transcription to produce precursor CRISPR RNA (pre-crRNA), which subsequently undergoes processing to yield crRNAs carrying a sequence complementary to the foreign genes. Upon recognizing sequences homologous to the viral gene, the crRNAs guide Cas nucleases to bind to and cleave the target gene, protecting the bacterial host from viral infiltration (36).

Based on differences in the sequences and structures of Cas proteins, CRISPR/Cas systems can be classified into two classes and six types (37). The class 1 CRISPR/Cas system contains a multisubunit crRNA–effector complex, including the type I, type III and type IV CRISPR/Cas system (38). However, the class 2 CRISPR–Cas system which includes the type I, type III and type IV CRISPR/Cas system just needs a single subunit crRNA–effector module (38). The detailed description of these CRISPR/Cas system is provided in the **Table 1**. As the simple and flexible genome editing method, the CRISPR/Cas system has revolutionized the biomedical research field (39). The type II CRISPR/Cas system emerged as an simple and efficient genome editing tools for various cells and organisms (40, 41), only requiring a multi-functional Cas9 protein to interfere with the invading genetic elements (42). The type II CRISPR system provided the foundation for the CRISPR/Cas9 gene editing system. However, the efficiency of the CRISPR/Cas9 system in eukaryotes is influenced by the chromosomal status of the target site (6). To efficiently deliver the Cas9 protein into mammalian nuclei, its N- or C-terminus is fused with the eukaryotic nuclear localization signal (43).

**Table 1:** The summarization of all types of CRISPR/Cas systems.

Types	Subtypes	Class^*^	Main Features
I: Cascade effector complexes	I-A, I-B, I-C, I-D, I-E, I-F1, I-F2, I-F3	1	Effector modules composed of multiple Cas proteins, unique signature protein: Cas3 (38).
II: Cas9 effector protein	II-A, II-B, II-C1, II-C2	2	A single subunit crRNA–effector module, effector protein: Cas9 (37).
III: Csm (III-A,D,F,E) and Cmr (III-B,C) effector complexes	III-A, III-B, III-C, III-D, III-E, III-F	1	Effector modules composed of multiple Cas proteins, unique signature protein: Cas10 (38).
IV: Csf effector complexes and IV-C effector complex	IV-A, IV-B, IV-C	1	Typically lack both adaptation modules and the necessary nucleases for interference (37). May be highly divergent derivatives of type I or type III.
V: Cas12 effector protein	V-A, V-B1, V-B2, V-C, V-D, V-E, V-F1, V-F1(V-U3), VF2, V-F3, V-G, V-U1, V-U2, V-U4, V-K(V-U5)	2	A single subunit crRNA–effector module, effector protein: Cas12 (37).
VI: Cas13 effector protein	VI-A, VI-B1, VI-B2, VI-C, VI-D	2	A single subunit crRNA–effector module, effector protein: Cas13 (37).

*Class 1 CRISPR–Cas systems are characterized by effector modules consisting of multiple Cas proteins, forming a crRNA-binding complex. These components function collaboratively in the binding and processing of the target. Class 2 systems feature a singular, multidomain crRNA-binding protein that serves a functional role analogous to the entire effector complex found in Class 1.

**Figure 2:**
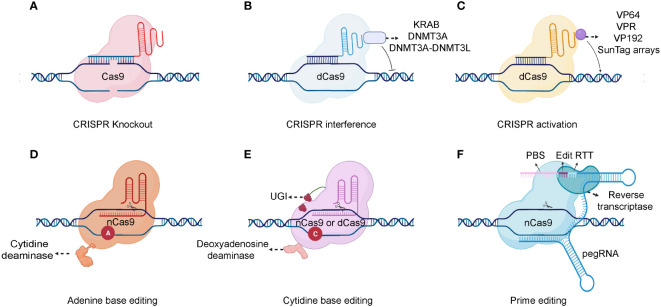
Schematic of different CRISPR-Cas system types. **(A)** A gRNA is used to identify the target genome sequence, guiding the Cas9 endonuclease to precisely cut the DNA double-strand. **(B)** For gene repression, dCas9 can be fused to KRAB, DNMT3A, or DNMT3A-DNMT3L. **(C)** Various transcription activators can be fused to dCas9, including VP64, VP192, SunTag arrays, and the VPR comprising VP64, p65, and Rta. **(D, E)** Combining dCas9 or nCas9 with cytidine or adenine deaminases makes it possible to directly convert cytidine into uridine or adenine into inosine. **(F)** As a fusion protein, prime editor is composed of reverse transcriptase and nCas9. Prime editor binds to a specific target DNA sequence under the guidance of prime-editing guide RNA (pegRNA). And a single-strand break is made by the nCas9. There are two main components of pegRNA: the reverse transcription template (RTT) and the protospacer binding sequence (PBS). During reverse transcription, the RTT encodes the desired edits which are primed by PBS and the edits are incorporated into the newly synthesized DNA strand. After DNA repair, the edits are stably incorporated into the genome. Created with BioRender.com.

The CRISPR/Cas9 system comprises two primary components: a single-stranded guide RNA (sgRNA) and a Cas protein. A trans-activating crRNA (tracrRNA) and a crRNA are fused to form a sgRNA. CRISPR/Cas9 technology uses the sgRNA to identify the target genome sequence, guiding the Cas9 endonuclease to precisely cut the DNA double-strand (44). Gene knockout or knock-in events occur during the repair process. Typically, cells use highly efficient non-homologous terminal junctions to repair broken DNA. During the repair process, base insertions or deletions often lead to frameshift mutations, knocking out the target gene (**Figure 2A**) (45). After a DNA double-strand break, if a DNA repair template enters the cell, the broken part of the gene will undergo homologous recombination repair using the repair template, causing gene knock-in (46).

The Cas9 nickase (nCas9) and dead Cas9 (dCas9) are also applied in the CRISPR based genetic modification. In detail, the nCas9 is a Cas9 variant protein that generated from the mutation in either RuvC nuclease domain or histidine/asparagine (HNH) endonuclease domain (47). While, the dCas9 is another variant Cas9 that generated from the mutation in both endonuclease domains (RuvC nuclease domain and HNH endonuclease domain) (48). Consequently, nCas9 can only induce a single-strand nick in the target DNA rather than a DNA double-strand break (DSB). And, the dCas9 specifically binds to the target gene guided by the sgRNA but lacks DNA cleavage activity. The binding of dCas9 to a gene’s transcriptional start site can obstruct its transcription initiation, impeding its expression (49). The dCas9 protein can also interact with transcriptional suppressors or activators to inhibit or activate downstream target gene transcription (50) (**Figures 2B, C**). Additionally, combining dCas9 or nCas9 with cytidine or adenine deaminases enables the direct conversion of cytidine into uridine or adenine into inosine (51, 52)(**Figures 2D, E**). Although base editing can introduce point mutations efficiently, it is difficult to generate precise indels and avoid bystander mutations (53). A recent genome editing technology called prime editing allows point mutations, small insertions, and small deletions to be introduced precisely and effectively with a favorable editing to indel ratios (54) (**Figure 2F**). Prime editor is composed of reverse transcriptase and nCas9. Prime editor binds to a specific target DNA sequence under the guidance of prime-editing guide RNA (pegRNA) and a single-strand break is made by the nCas9. During a reverse transcription, the desired edits are encoded and are incorporated into the newly synthesized DNA strand. After DNA repair, the edits can be stably incorporated into the genome. In conclusion, the CRISPR/Cas9 system is already significantly contributing to functional genomic experiments and is poised to have an unprecedented impact on experimental biology in the future (55). Moreover, it holds immense potential for diverse gene therapy applications, including blood diseases, tumors, and other genetic disorders.

## 4 High-throughput CRISPR technology

### 4.1 CRISPR screening

Genetic screening is a powerful tool for investigating genes contributing to diverse biological phenotypes and diseases. Numerous genome-wide targeted techniques have emerged with advancements in genetic tools. For example, RNAi libraries were commonly used to screen gene functions due to their efficiency in reducing gene expression at the mRNA level (56). RNAi has been the favored approach for studying gene function in the last decade, particularly in functional genomic screening (6). However, CRISPR has emerged as a more powerful genome editing technique than RNAi, with lower off-target efficiency and higher interference levels (6). Studies have used Cas9 to perform experiments with remarkable results in various cell lines and animal models. However, using one or more sgRNAs for gene knockdown lacks sufficient throughput. Therefore, CRISPR screening was developed based on the RNAi screening technique. Compared to RNAi-based screenings, CRISPR screening provides higher validation rates, greater phenotypic effects, minimal off-target effects, and consistent results (7, 8). Moreover, the comparison between CRISPR based screening and RNAi based screening are detailed described in **Table 2**.

**Table 2:** The comparison between CRISPR based screening and RNAi based screening.

	RNAi screening	CRISPR screening	Reference
Mechanism of action	(1) Perturbation occurs in the cytoplasm (Gene perturbation is not biased by cell ploidy, chromatin conformation or locus accessibility). (2) Regulating genes at mRNA or non-coding RNA level. (3) Simple: RNAi screening does not require co-delivery of exogenous proteins or introduce foreign sequences encoding large proteins.	(1) Perturbation occurs in the nucleus (Gene perturbation is mainly biased by cell ploidy, chromatin conformation or locus accessibility). (2) Regulating genes at the genomic DNA level. (3) Complex: CRISPR Screening need co-delivery or introduce foreign sequences of Cas 9 protein.	(6, 57)
Efficiency	Low	High	(3, 58)
Off-target effects	High	Low	(6)
Reversibility of perturbation	Reversible	Reversible or Irreversible	(57)
Main systems	shRNA	CRISPR activation, CRISPR interference, and CRISPR Knockout	(59, 60)

#### 4.1.1 CRISPR screening workflow

**Figure 3:**
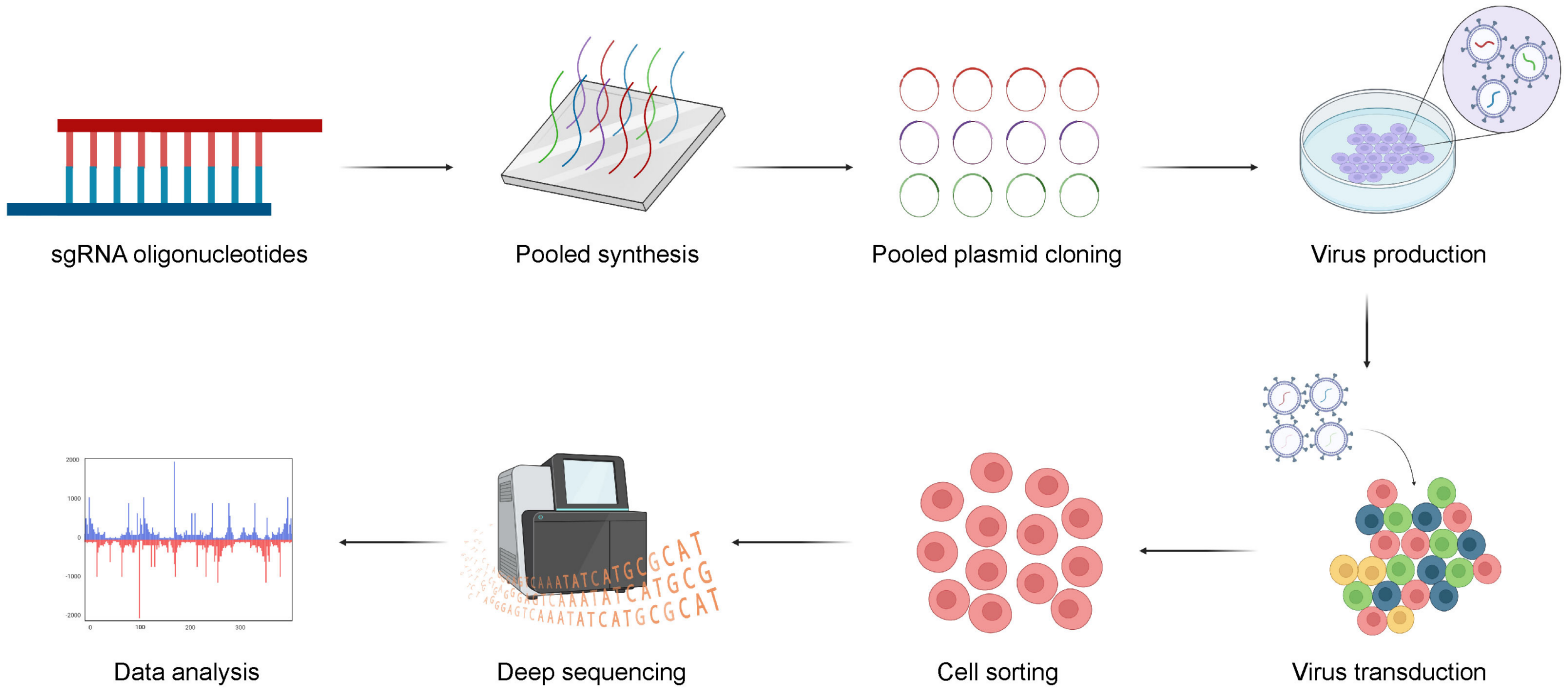
The general workflow of CRISPR screening. Firstly, three or more sgRNAs are designed for each gene, sgRNAs are synthesized with high throughput and cloned into a lentiviral vector. And, the lentiviral library is transduced into target cells and the transduced cells are sorted for sequencing with multiple selective strategies. Finally, the potential genes can be validated with bioinformatic analysis. Created with BioRender.com.

The general CRISPR screening workflow can be summarized in the following four steps (61) (**Figure 3**). Firstly, three or more sgRNAs are designed for each gene in a species, which are synthesized with high throughput and cloned into a lentiviral vector. Subsequently, the lentiviral library is packaged with a low multiplicity of infection (MOI; generally <0.3) to infect target cells, ensuring that each cell is infected by only one viral particle. The entire transduced cell pool is divided into two groups. One half serves as the experimental group and is subjected to screening pressure, such as viral infection or drug treatment, while the other serves as the control group. Cells are sorted based on phenotypic traits such as drug resistance, proliferation capacity, and survival capability. Finally, genomes of sorted cells are isolated from both the experimental and control groups. Then, sgRNA fragments are polymerase chain reaction (PCR) amplified, high-throughput sequenced, and bioinformatically analyzed.

#### 4.1.2 Phenotypic selection in CRISPR screening

CRISPR screening is primarily used to screen functional genes. Depending on the specific screening objectives, it can be categorized into positive and negative selection. Positive selection involves applying selective pressure to a cell library with successfully integrated sgRNA to promote the survival of cells with the desired phenotypes and ultimately enrich key genes (62). Conversely, negative selection involves the survival of cells not exhibiting the desired phenotypes. Determining sgRNA abundances at different time points allows for identifying differences in sgRNA profiles, revealing key genes (57). Negative screening can identify genes responsible for inducing specific functional deficits in cells (63). For example, essential genes required for cell survival can be identified by extending the screening duration. Different cellular phenotypes can also be reflected by fluorescent signals and sorted by fluorescence-activated cell sorting (FACS) (64). The potential targets controlling specific cellular phenotypes can then be identified by deep sequencing. Moreover, the cells for sequencing can also be sorted using magnetic-activated cell sorting (MACS) based on their expression of a specific surface protein (64).

#### 4.1.3 Design tools for sgRNAs

##### 4.1.3.1 CrisprVerse: A new ecosystem of R packages

Various CRISPR technologies, including knockout or knockdown, activation or interference, and base editing, significantly enhance gRNA design and annotation capabilities (65). The core R package, *crisprDesign*, offers a user-friendly and standardized interface that supports adding off-target annotations, comprehensive gene annotations, and assessments of both on- and off-target activity ratings (65). All nucleases targeting DNA or RNA can access these features, including Cas9, Cas12, and Cas13 (65).

##### 4.1.3.2 CRISPOR

CRISPOR.org is a web-based tool designed explicitly for CRISPR/Cas9 genome editing research. This platform identifies gRNAs within an input sequence and uses various metrics to rank them, evaluating their potential for off-target effects in the target genome and predicting their on-target activity (66). Recent advancements in CRISPOR include the development of next-generation sequencing primers for off-target mutation assessment, customized oligonucleotide creation for guide cloning, and batch design strategies for genome-wide CRISPR screening (66).

##### 4.1.3.3 The UCSC genome browser

The University of California-Santa Cruz (UCSC) genome browser provides diverse information and tools that enhance our understanding of the genomic context across numerous species. It facilitates in-depth data analysis and enables queries of regions of interest within diverse datasets from multiple sources (67). In addition, researchers can include their data in the primary display, facilitating data querying and sharing with others (67). When conducting CRISPR/Cas9 genome editing research, one can select from a pool of reliable gRNA sequences previously generated by focusing on the specific target gene region (68).

##### 4.1.3.4 E-CRISP

E-CRISP is a web application developed for designing genomic RNA sequences that provides experiment-centered design parameters, enabling the construction of multiple libraries and the systematic investigation of the effects in diverse settings (69). E-CRISP identifies target sequences that are complementary to the gRNA and terminate with the N(G) or A(G) protospacer-adjacent motif (PAM). This motif is essential for enabling the Cas9 nuclease to cleave the DNA double-strand upon activation (69). E-CRISP uses a binary interval tree to rapidly annotate potential gRNA target positions and a fast-indexing strategy for locating binding sites (69).

##### 4.1.3.5 CHOPCHOP

CHOPCHOP is a web-based tool to identify sgRNA targets within the CRISPR/Cas system (70). Its guiding principle is to provide an accessible and powerful tool to benefit users of all levels, whether novice or experienced (71). CHOPCHOP provides various advanced options for selecting targets and accommodates various inputs, such as gene IDs, genomic regions, or pasted sequences (72). It applies efficient sequence alignment techniques to reduce search times and duplication by accurately predicting the off-target binding of sgRNAs (72). Each query generates an interactive representation of the gene, displaying potential target sites at their genomic locations, color-coded based on their quality ratings (72).

##### 4.1.3.6 CRISPRscan

The variability in activity among numerous sgRNAs remains a significant problem (73). Moreno-Mateos et al. discovered that increasing guanine and decreasing adenine content improved sgRNA stability and activity (73). They also found that sgRNAs with 5’ mismatches and 1–2 nucleotide truncations could effectively replace conventional sgRNAs (73). Based on their findings, they developed the CRISPRscan predictive sgRNA-scoring algorithm, which effectively captures the sequence attributes that impact CRISPR/Cas9 *in vivo* activity (73).

##### 4.1.3.7 GuideScan

GuideScan is a serial software that is computationally intensive in building CRISPR guide RNA libraries from big genomes (74). GuideScan generates high-density sets of single- and paired-gRNAs for genome-wide screening (75). GuideScan precisely and efficiently tallies all targetable sequences within a specified genome using a retrieval tree data structure (75). Additional details regarding each gRNA, including its on-target efficiency scores and overlapping genomic features, can be incorporated into its annotation (75).

#### 4.1.4 Analysis tools for CRISPR screening

In this section, we discuss the current analysis tools for CRISPR screening. Moreover, we provide the detailed information of analysis tools in **Table 3**.

**Table 3:** Tools for CRISPR screens results analysis.

Method	Description	Language	Visualization Tools
MAGeCK	MAGeCK outperforms existing computational methods in its control of the FDR and its high sensitivity, identifies both positively and negatively selected genes simultaneously, and reports robust results across different experimental conditions (76).	Python,R	Yes
MAGeCK-VISPR	MAGeCK-VISPR overcomes these computational limitations of the CRISPR screening. It defines a set of QC measurements and can call essential genes under multiple conditions meanwhile considering sgRNA knockout efficiency (77).	Python,R	Yes
MAGeCK Flute	MAGeCK Flute is an algorithm that combines the MAGeCK and MAGeCK-VISPR algorithms (78).	Python,R	Yes
BAGEL	BAGEL is a supervised learning method for analyzing gene knockout screenings coupled with gold-standard reference sets of essential and nonessential genes (79).	Python	No
CERES	CERES can analyze gene-dependency levels as well as estimate the copy number specific effect (80).	R	No
JACKS	JACKS is a Bayesian method that can use the same gRNA library to model gRNA efficacies in multiple screenings (81).	Python	No

##### 4.1.4.1 MAGeCK

While not designed explicitly for CRISPR/Cas9 knockout screenings, several established algorithms can still be used to identify significantly enriched sgRNAs or genes in next-generation RNA sequencing, genome-scale small interfering RNA, or shRNA screens (76). The Model-based Analysis of Genome-wide CRISPR/Cas9 Knockout (MAGeCK) method surpasses current computational methods in controlling the false discovery rate and shows high sensitivity. It concurrently identifies genes selected in both positive and negative directions, providing consistent outcomes across various experimental conditions (76). Distinct MAGeCK algorithms typically comprise two key steps (1): Analyzing raw read counts associated with sgRNAs by median normalization and (2) modeling the mean-variance relationship within replicates using a negative binomial distribution to assess the differences between treatments and controls (82).

##### 4.1.4.2 MAGeCK-VISPR

High-throughput sequencing data can only be analyzed by comparing two conditions in most algorithms developed for screening analysis. However, in many cases, screenings are conducted simultaneously across various time points, treatment conditions, or cell lines (77). MAGeCK-VISPR overcomes these computational limitations of CRISPR screening by defining a set of quality control (QC) measurements. In order to iteratively estimate gene essentiality and sgRNA knockout efficiency, the algorithm uses a generalized linear model to deconvolute different effects. This method can identify essential genes under different conditions while considering the sgRNA knockout efficiency (77). In addition, it provides a web-based visualization framework to allow users to interactively explore CRISPR screening QC and analysis results (77).

##### 4.1.4.3 MAGeCKFlute

The MAGeCKFlute algorithm combines the MAGeCK and MAGeCK-VISPR algorithms and offers downstream analysis capabilities (78). MAGeCKFlute includes several functions, including normalization, batch effect removal, and QC (78). A desktop computer with Linux or Mac OS and support for R is sufficient to complete the whole MAGeCKFlute pipeline in approximately two hours. MAGeCKFlute’s comprehensive pipeline includes various modules for analyzing CRISPR screening data, making it unique among competing tools.

##### 4.1.4.4 BAGEL

The Bayesian Analysis of Gene EssentiaLity (BAGEL) method is executed as a Python script, requiring the Python modules *numpy* and *scipy*. A gold-standard reference set of essential and non-essential genes is used to analyze gene knockout screens (79). Furthermore, BAGEL2 was developed in 2021, which can detect tumor suppressor genes and uses a multi-target correction to mitigate false positives resulting from off-target CRISPR gRNAs (83). BAGEL2 shows enhanced sensitivity and specificity compared to BAGEL.

##### 4.1.4.5 CERES

The sgRNA-Cas9 complex can target the genomic copy number to affect cell proliferation in genome-scale CRISPR/Cas9 loss-of-function screens (8, 84) mainly through the independent antiproliferative effect of Cas9-mediated DNA cleavage. The CRISPR Engineering for the Rapid Enhancement of Strains (CERES) method enables the analysis of gene-dependency levels and estimation of the copy number-specific effect (80). Moreover, Tsherniak et al. found it could decrease false positives in CRISPR-Cas9 essentiality screens of cancer cell lines (80).

##### 4.1.4.6 JACKS

The signal variability caused by different gRNAs targeting the same gene limits the applications of genome-wide CRISPR/Cas9 knockout screens (81). Joint Analysis of CRISPR/Cas9 Knockout Screens (JACKS) uses a Bayesian approach to model gRNA efficacies across multiple screens using the same gRNA library (81).

### 4.2 Perturb-seq: combining CRISPR screening and emerging single-cell technologies

Analyzing gene and cellular functions by establishing connections between genetic changes and their phenotypic outcomes is of paramount significance (85). While genetic screenings are powerful tools that can help us identify the inferred gene function in mammalian cells, they cannot effectively investigate complex phenotypes, such as transcription profiles (86). Such screenings focus on individual perturbations, delivering and evaluating them separately (86).

Genetic perturbation has emerged as a tool to elucidate causal connections between genes and phenotypes (87). With the help of perturb-seq, pooled CRISPR screens can be combined with single-cell RNA sequencing to investigate functional CRISPR screens at a single-cell level (87). Additionally, genome-scale Perturb-seq can multi-dimensionally reveal cellular behavior, gene function, and regulatory networks. We can also use the Perturb-seq to obtain rich genotypic and phenotypic maps, realizing systematic genetic and cellular function exploration. For example, Zhang et al. used Perturb-seq *in vivo* to determine the relationships of autism risk genes with neuronal and glial abnormalities (88). Moreover, Hein and Weissman used Perturb-seq to characterize thousands of CRISPR-modified cells, revealing the roles of host and viral factors (89). Moreover, a more powerful genome-scale Perturb-seq method was developed in 2022, which uses CRISPR interference (CRISPRi) to target all expressed genes across >2.5 million human cells (85).

In conclusion, we can obtain rich biological insights and identify the mechanisms of complex biological responses using Perturb-seq (90). Perturb-seq allows us to extract additional experimental data, contributing to the advancement of biomedical research.

## 5 CRISPR screening in immune cells

**Figure 4:**
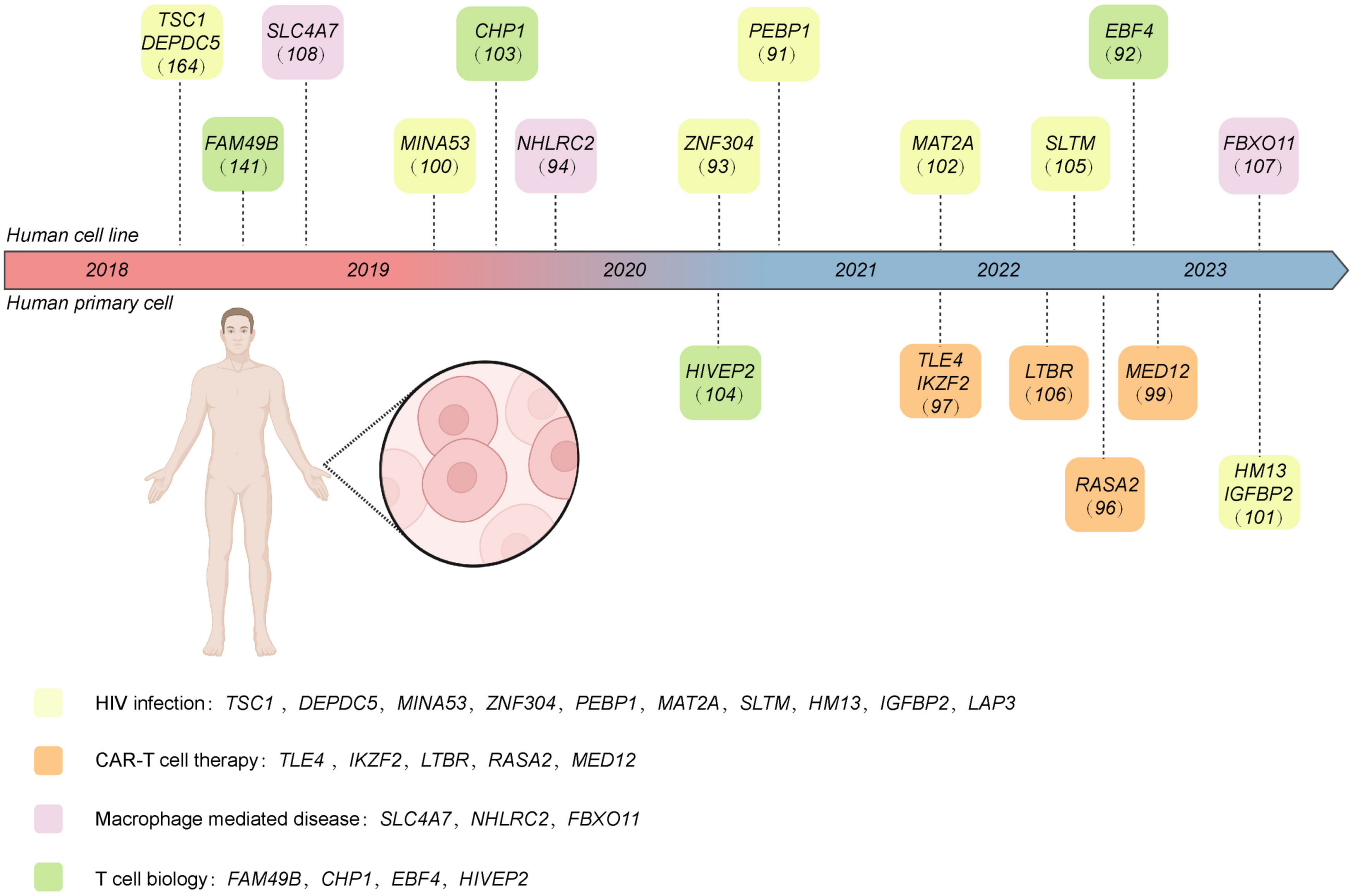
The target genes of CRISPR screening performed in human-derived immune cells discussed in this review. These genes are classified into different research areas, including CAR T therapy, HIV infection, macrophage-mediated disease, and T cell biology. Created with BioRender.com.

In this section, we discuss current CRISPR screening in immune cells, with a particular focus on human or mouse-derived immune cells. Moreover, we provide a summarization in **Tables 4** and **5**. We also systematically summarize the different response genes identified in human and mouse-derived immune cells using CRISPR screening in **Figure 4** and **Table 6**.

**Table 4:** CRISPR screening studies in human immune cells.

Name of library	Library Type	Library Size	Cell type	Screening methods	Reference
Human CRISPR Knockout Pooled Library (GeCKO v2)	Knockout	Genome-wide	C11 (CD4^+^ T model of HIV latency)	FACS (Sorting GFP^-^ and GFP^+^ cells for sequencing)	(91)
Human CRISPR Knockout Pooled Library (GeCKO v2)	Knockout	Genome-wide	Jurkat cell	The transduced cells were divided into control and FASL stimulated groups, and then the cells were sequenced	(92)
Human CRISPR Knockout Pooled Library (GeCKO v2)	Knockout	Genome-wide	Jurkat cell	FACS (sorting BFP^-^ and BFP^+^ cells for sequencing)	(93)
Human CRISPR Knockout Pooled Library (GeCKO v2)	Knockout	Genome-wide	THP1 monocyte	FACS (sorting GFP^-^ macrophages for sequencing)	(94)
Human CRISPR Knockout Pooled Library (GeCKO v2)	Knockout	Genome-wide	J-Lat 10.6 cell	FACS (Sorting GFP^-^ and GFP^+^ cells for sequencing)	(95)
Human CRISPR Knockout Pooled Library (Brunello)	Knockout	Genome-wide	Primary human T cell	FACS (The CFSE ^high/low^ cells were selected and sequenced separately)	(96)
Human CRISPR Knockout Pooled Library (Brunello)	Knockout	Genome-wide	Primary human T cell	FACS (PD1^-^ and PD1^+^ CAR T cells were selected for sequencing)	(97)
Human CRISPR Knockout Pooled Library (Brunello)	Knockout	Genome-wide	Primary human T cell	FACS (sorting of cells for sequencing based on CFSE)	(98)
Bassik Human CRISPR Knockout Library	Knockout	Genome-wide	Primary human T cell	FACS (sorted for high cytokine expression)	(99)
Human CRISPR Knockout Pooled Libraries (Enriched Sub-pools)	Knockout	Genome-wide	J-LAT A2 cell	FACS (Sorting GFP^-^ and GFP^+^ cells for sequencing)	(100)
CD4-ISG sgRNA library	Knockout	Custom	Primary human T cell	After transduction, the cells were treated with IFN for 24h, then infected with HIV, and viral RNA and cellular DNA were extracted for sequencing	(101)
human CRISPR metabolic gene knockout library (MeGKO library)	Knockout	Custom	J-Lat 9.2	FACS (Sorting GFP^-^ and GFP^+^ cells for sequencing)	(102)
metabolism focused library	Knockout	Custom	Jurkat cell	The transduced cells were divided into two groups, one group without any treatment and one group with Palmitate treatment	(103)
Pooled RNP library	Knockout	Custom	T regulatory cell	FACS (Cells were sorted according to Treg cell markers (FOXP3 and CTLA-4), proinflammatory effector cytokine (IFN-γ) for sequencing)	(104)
Human Genome-wide CRISPRi-v2 Libraries	Inhibition	Genome-wide	Jurkat cell	FACS (Sorting of HIV-1-GFP^high^ cells and other cells (control) for sequencing)	(105)
Lentiviral genome-scale barcoded ORF library	Activation	Custom	Primary human T cell	FACS(Cells were sorted according to the level of CFSE)	(106)
Brunello CRISPR knockout library	Knockout	Genome-wide	THP-1 monocyte	FACS (sorting Viable macrophages for sequencing)	(107)
SLC-wide knockout library	Knockout	Custom	U937 cell	FACS (sorting Phagocytosis positive (PhagoLate) and phagocytosis negative (PhagoNeg) populations)	(108)

**Table 5:** CRISPR screening studies in murine immune cells.

Name of library	Library Size	Cell type	*In vivo* or *in vitro*	Screening methods	Reference
Mouse CRISPR Knockout Pooled Library (Brie)	Genome-wide	Naïve CD4^+^ T cell	*In vitro*	FACS (Sorting of BFP^+^ IL-13Tom^+^ and BFP^+^ IL-13Tom^-^ cells for sequencing)	(109)
Mouse CRISPR Knockout Pooled Library (Brie)	Genome-wide	Naïve CD4^+^ T cell	*In vitro*	FACS (Sorting of BFP^+^ IL-13Tom^+^ and BFP^+^ IL-13Tom^-^ cells for sequencing)	(110)
Mouse CRISPR Knockout Pooled Library (Brie)	Genome-wide	T regulatory cell	*In vitro*	FACS (sorting of Foxp3^hi^ and Foxp3^lo^ for sequencing)	(111)
Mouse CRISPR Knockout Pooled Library (Brie)	Genome-wide	CD8^+^ T cell	*In vivo*	The transduced cells were transfused into B16-OVA-melanoma bearing mice, and then the OT-1 TILs were extracted.	(112)
Mouse CRISPR Knockout Pooled Library (Brie)	Genome-wide	CD4^+^ T cell	*In vitro*	FACS (sorting of (p-S6hi) and (p-S6lo) cells for sequencing)	(113)
Mouse CRISPR Knockout Pooled Library (Brie)	Genome-wide	BMDMs	*In vitro*	FACS (sorting of non-eaters and efficient eaters for sequencing)	(114)
Mouse CRISPR Knockout Pooled Library (Brie)	Genome-wide	iBMM	*In vitro*	FACS (sorting of phagocytosis-deficient cells for sequencing)	(115)
Mouse CRISPR Knockout Pooled Library (Brie)	Genome-wide	B cell	*In vitro*	FACS (sorting of GFP^+^, mCherry^+^ IgE^+^ iGCs, IgG1^+^ iGCs, IgE^+^ PCs, and/or IgG1^+^ PCs for sequencing)	(116)
Mouse CRISPR Knockout Pooled Library (Brie)	Genome-wide	CD8^+^ T cell	*In vivo* and *in vitro*	Transduced cells are transfused into C57BL/6 mice and then Thy1.1^+^ cells are sorted from the spleen (*in vivo*) or cultured *in vitro* (*in vitro*).	(117)
Teichmann Retroviral Mouse Genome-wide CRISPR Knockout Library	Genome-wide	CD8^+^ T cell	*In vivo*	FACS (the cells were divided into Chronic Stim (IL-2^+^αCD3) and Acute Stim (IL-2 only), followed by sequencing)	(118)
Genome-scale mouse T cell CRISPR library (MKO)	Genome-wide	CD8^+^ T cell	*In vivo*	The transduced cells were transfused into Rag^-/-^mice bearing E0771-mCh-OVA tumor, and then TILs were extracted for sequencing.	(119)
Teichmann Retroviral Mouse Genome-wide CRISPR Knockout Library	Genome-wide	naïve CD4^+^ T Cell	*In vitro*	FACS (cells were sorted and sequenced according to Il4, Il13, Xbp1, and Gata3)	(120)
Mouse Improved Genome-wide Knockout CRISPR Library v2	Genome-wide	CD4^+^ T cell	*In vitro*	FACS (Sorting of CFSE^hi^ and CFSE^lo^ Cas9-CD45.1 CD4^+^ T cells for sequencing)	(121)
PIP3-binding proteins CRISPR library	Custom	CD8^+^ T cell	*In vitro*	FACS (Sorting of scICAM-1neg (not binding ICAM-1) and scICAM-11pos (ICAM-1-binding) GFP^+^ cells for sequencing)	(122)
25 kinases library	Custom	CD8^+^ T cell	*In vitro*	FACS (ROS downregulated and γH2AX downregulated were sorted out by FACS and sequenced.)	(123)
Lentiviral sgRNA metabolic library	Custom	SMARTA cell	*In vivo*	FACS (TH1 and TFH cells were sorted out based on SLAM and CXCR5 indexes.)	(124)
Lentiviral sgRNA metabolic library	Custom	CD8^+^ T cell	*In vivo*	The transduced cells were transfused into B16-OVA-melanoma bearing mice, the OT-1 TIL extracted after transfection.	(112)
Lentiviral sgRNA metabolic library	Custom	CD8^+^ T cell	*In vivo*	FACS (Sorting of KLRG1^-^CD127^+^ and KLRG^+^CD127^-^ cells for sequencing)	(125)
membrane bound protein gene targeting single-strand RNA (sgRNA) library	Custom	Naïve CD8^+^ T cell	*In vivo*	After transduction, cells were transfected to GBM-bearing mice, and then CD8^+^ from brain tumor tissue was extracted for sequencing.	(126)
120-TF targeting sgRNA library	Custom	CD8^+^ T cell	*In vivo*	The sgRNA^+^Cas9^+^ CD8 T cells extracted after transfection were sequenced with the sgRNA^+^Cas9^+^ CD8 T cells before transfection.	(127)
1C metabolism gRNA library	Custom	CD4^+^ T cell	*In vivo*	The transduced cells were transfused to LCMV infected mice, the sgRNA^+^ Cas9^+^ CD8 T cells extracted after transfection.	(128)
Retroviral sgRNA epigenetic library	Custom	CD8^+^ T cell	*In vivo*	FACS (cells are sorted according to KLRG1 and CD127, and sequenced.)	(129)
genome-scale catalytically dead guide RNA (dgRNA) library	Genome-wide	CD8^+^ T cell	*In vitro*	FACS (Sorting of CD8^+^ CD107a^+^ cells for sequencing)	(130)
RBP sgRNA library	Custom	Raw 264.7 cell	*In vitro*	FACS (the cells for sequencing sorted by flow cytometry on the basis of the expression levels of TNF-α)	(131)

**Table 6:** The therapeutic targets in murine immune cells identified by CRISPR screening.

Immune cell type	Cell phenotype	Therapeutic targets	Function	Clinical application	Reference
CD4+T cell	TFH cell differentiation	*Etnk1、Pcyt2、SelenoI*	Metabolism	Therapeutic strategy to treat autoimmunity	(124)
	Naïve T helper cell→T helper type 2	*Pparg、Bhlhe40*	Metabolism	Therapeutic strategy to treat infection, autoimmunity, and tumor immunology	(120)
	Foxp3 expression	*Brd9*	Epigenetics	Therapeutic strategy to treat autoimmunity and cancer	(111)
	Effector and Regulatory T Cell Fate	*Mthfd2*	Metabolism	Therapeutic strategy to treat CD4^+^ T-cell driven inflammation	(128)
	T cell priming and Treg suppressive function	*Sec31a*	Metabolism	Therapeutic strategy to treat infectious diseases and other immune-mediated disorders	(113)
	CD4^+^ T cell recruitment and proliferation	*Socs1*	Cytokine signaling	CAR-T cell therapy	(121)
	T helper 2 immune cell differentiation	*Adnp*	Epigenetics	Therapeutic strategy to treat allergic disease	(109)
	TH2 cell differentiation	*Itgav, Itgb3*	Checkpoint	Therapeutic strategy to treat chronic inflammatory diseases	(110)
CD8+T cell	T cell exhaustion	*Ino80, Baf*	Epigenetics	Therapeutic strategy to treat cancer	(118)
	Anti-tumor T cells: expansion, differentiation, oxidative stress, and genomic stress	*Mapk14*	Metabolism	Adoptive Immunotherapies	(123)
	CD8^+^T cell: effector functions, cytokine production, and T cell activation	*Dhx37*	RNA helicase	Therapeutic strategy to treat cancer	(119)
	T cell migration, homeostasis, and function	*Rasa3*	TCR signal	Therapeutic strategy to treat immune-mediated disease	(122)
	T cell effector functions	*Pdia3*	TCR signal	Glioblastoma immunotherapy	(126)
	T-cell persistence and effector function	*Batf*	Epigenetics	Cancer immunotherapy	(112)
	CD8^+^ T cell fate	*Slc7a1, Slc38a2*	Metabolism	Therapeutic strategy to treat cancer and infection	(125)
	Effector T cell (Teff) differentiation	*Fli1*	Epigenetics	Therapeutic strategy to treat cancer and infection	(127)
	T-cell effector function	*Prodh2*	Metabolism	CAR-T therapy	(130)
	CD8^+^ T cell fate	genes of cBAF	Epigenetics	CAR-T therapy	(129)
B cell	B cell fate	*Prc2*	Epigenetics	Targeted therapeutics to treat immune disorders and hematological malignancies	(132)
	Plasma cell differentiation	*Blimp1*	Epigenetics	Therapeutic strategy to treat those autoimmune diseases	(133)
	Memory B cells differentiation	*Hhex*	Transcription factors	Inducing long-lasting protective immunity	(134)
	Plasma cell differentiation	*Pi3k*	Metabolism	Therapeutic strategy to treat allergic disease	(116)
Macrophage	Myeloid restriction of Listeria monocytogenes infection	*Pten*	Protein and lipid phosphatase	Therapeutic strategy to treat Listeria monocytogenes infection	(115)
	Macrophage recognition of fungi	genes of C3aR	G-protein coupled receptor	Therapeutic strategy to treat fungi infection	(135)
	parasite fitness in naїve or IFNγ-activated murine macrophages	*Gra45*	ASP5 substrate	Therapeutic strategy to treat parasite infection	(136)
	Macrophage Viability and Inflammatory	*Tnf*	Pro-inflammatory cytokine	Presenting evidence for alternative therapeutic strategies	(137)
	Macrophage efferocytosis	*Wdfy3*	Phosphatidylinositol 3-phosphate-binding protein	Embryogenesis and development, and the resolution of pathological events	(114)
	Macrophage activation	*Mettl3*	Epigenetics	Cancer immunotherapy	(131)

### 5.1 CRISPR screening in various areas based on human immune cells

#### 5.1.1 Chimeric antigen receptor - T cell therapy

Chimeric antigen receptor (CAR)-T cell treatments have become a pivotal therapeutic approach for various hematological cancers (138). However, CAR-T cell therapy has various limitations, including antigen escape, off-target effects, CAR-T cell trafficking, tumor infiltration, and CAR-T cell-associated toxicity (139). Genome-scale CRISPR screenings in primary human T cells can comprehensively explore potential targets in CAR-T therapy (98). However, conducting CRISPR screens in primary human cells has been challenging. In 2018, Marson et al. introduced a novel method called sgRNA lentiviral infection with Cas9 protein electroporation (SLICE) that can precisely and efficiently disrupt target genes in primary human cells (98). They introduced the Brunello human CRISPR knockout pooled library into primary human T cells using the SLICE method (96). The transduced cells were cultured under a series of immunosuppressive conditions to identify genes causing T cell dysfunction in the tumor microenvironment (TME) (96). Their findings suggest that RASA2 is a prospective target for improving T cell therapy effectiveness and persistence in treating cancer.

In addition, inadequate T cell potency is a significant obstacle to T cell immunotherapy progress (99). Recently, Mackall et al. used the Bassik human CRISPR knockout library to target primary T cells, which were then transformed into CAR-T cells and co-cultured with tumor cells (99). *MED12* deletion enhanced CAR T cells’ antitumor ability and sustained effector phenotype. Moreover, glioblastoma multiform (GBM), a primary brain tumor, is characterized by its uniform lethality and resistance to conventional treatments (140). However, CAR T cells show a limited general therapeutic effect in GBM. After transduction with a whole-genome knockout CRISPR/Cas9 library, CAR T cells were co-cultured with glioblastoma stem cells. Then, PD1^+^ and PD1^−^ CAR T cells were individually sorted and sequenced (97). Notably, *TLE4* and *IKZF2* were identified as potential targets, and their knockout enhanced CAR antitumor efficacy. Nevertheless, a major limitation of CRISPR-based loss-of-function screens is that they only focus on negative regulators of T-cell function and permanent genomic modifications. Sanjana et al. conducted a gain-of-function screening in primary human T cells using a gRNA library targeting human open reading frames (106). Overexpressing *LTBR* induced significant epigenomic and transcriptional remodeling, enhancing T cell effector capabilities and conferring resistance to exhaustion under chronic stimulation conditions.

#### 5.1.2 T cell biology

Despite decades of research on T-cell activation mechanisms, the exact mechanism remains elusive, posing a significant impediment to immunotherapy of T-cell tumors. Wang et al. conducted a genome-wide CRISPR screening in Jurkat cells. The transduced cells were sorted for sequencing based on their CD69 expression after T cell receptor (TCR) stimulation (141). FAM49B was found to prevent T cell activation by suppressing Rac activity and controlling cytoskeleton rearrangement.

Apoptosis, a programmed cell death mechanism in various cell types, is pivotal for immune system homeostasis, including removing activated immune cells (142). Interestingly, it was recently discovered that apoptosis is associated with the cytotoxic immune function of immune cells (143). In 2022, Lenardo et al. conducted a genome-wide CRISPR knockout screen for regulators of FAS/APO-1/CD95-mediated T cell death in Jurkat cells. The transduced cells were divided into control and Fas ligand (FASL)-stimulated groups (92). Their findings showed that EBF4 was indispensable for the cytotoxic functions of natural killer and CD8^+^ T cells by regulating TBX21, EOMES, granzymes, and perforin.

Moreover, increasing evidence shows that triglyceride and fatty acid metabolism are indispensable in regulating T cells (144). For example, exposure to circulating fatty acids can alter T cell function through metabolic reprogramming. In 2019, Birsoy et al. conducted a CRISPR knockout screen in Jurkat cells with two gRNA libraries: a metabolism-focused library and a genome-wide CRISPR library. The Jurkat cells were divided into control and palmitate-treated groups (103). Interestingly, they found that CHP1 regulated endoplasmic reticulum glycerolipid synthesis by binding and activating GPAT4 to catalyze the initial rate-limiting step in glycerolipid synthesis.

A key component of immune homeostasis and humoral immunity is the Treg (145). While the transcription factor (TF) FOXP3 is a known lineage-specific factor for Tregs, the key TFs controlling Treg gene expression are poorly understood (104). Marson et al. conducted a pooled and arrayed Cas9 ribonucleoprotein screen in primary human Tregs to identify the TFs involved in regulating critical proteins in human Tregs (104). Transduced cells were sorted for sequencing based on their protein expression under diverse cytokine conditions (104). Sequencing of single-cell transcriptomes revealed the distinct control of gene networks by FOXP3 and PRDM1. HIVEP2 was found to co-regulate a separate gene network with SATB1 in Treg cell-mediated immunosuppression.

#### 5.1.3 HIV infection

Antiretroviral therapy (ART) advancements have substantially reduced morbidity and mortality in individuals infected with the human immunodeficiency virus (HIV) (146). Individuals with HIV can effectively manage their infections over an extended period through daily pills or monthly injections (147). However, ART is not a curative method and must be taken for life. One of the major barriers to ART is the latent reservoir of HIV (148). HIV-1 preferentially infects the activated CD4^+^ T cells, and most infected cells die quickly (149). A few infected cells exist in a resting state, and the viral genome will integrate into their genome indefinitely (149).

Several inflammation-related genes are regulated by the canonical NF-κB and MAPK signaling pathways (150). In 2020, Zhu et al. conducted a genome-wide knockout screen in the C11 cell line using a human CRISPR knockout pooled library (GeCKOv2) (91). They discovered that PEBP1 could inactivate the MAPK/NF-κB signaling, inhibiting HIV transcription and inducing HIV latency. Moreover, ubiquitination is crucial in regulation of NF-κB signaling, such as ubiquitination mediated by E3 ubiquitin ligases (151). Ho et al. conducted a genome-wide knockout screen in the J-Lat 10.6 cell line using a human CRISPR knockout pooled library (95). The J-Lat 10.6 cell line contains an integrated replication-incompetent HIV-1 gene with a green fluorescent protein (GFP) reporter, with GFP expression correlating with HIV-1 reactivation (152). Combining the GFP expression and CRISPR screening results, they identified several deubiquitinases, including UCH37, USP14, OTULIN, and USP5, that may be HIV-1 latency regulators (95). This study was the first comprehensive investigation of HIV latency mechanisms centered on deubiquitinases.

Type I interferons (IFN-Is) are crucial in immune regulation during viral persistence, modulating various environmental and cellular functions (153). IFN-stimulated genes (ISGs) can prevent lab-adapted HIV infections in cells (153). However, their restrictive mechanism in primary CD4^+^T Cells, the HIV target cells, is poorly understood. Overbaugh et al. conducted a CRISPR-knockout screen in primary T cells using a CD4-ISG sgRNA library (101). They identified several previously unknown HIV-restrictive ISGs, including HM13, IGFBP2, and LAP3. This study represents one of the few articles investigating the HIV infection mechanism in primary T cells.

Several epigenetic regulators have also been found to contribute to HIV latency regulation through retrovirus and retrotransposon silencing (154–159). In 2019, Zhu et al. used a lentiviral sgRNA knockout sub-library to target nuclear proteins in J-LAT A2 cells hosting the latently infected “LTR-Tat-IRES-GFP” HIV-1 minigenome (100). The transduced cells were selected for sequencing based on their GFP expression level. They found that knockdown of the *MINA53* gene can reactivate the latent HIV consistently. Furthermore, Ho et al. conducted a CRISPRi screen in Jurkat cells using human genome-wide CRISPRi-v2 libraries. They found that *SLTM* knockdown promoted the reactivation of latent HIV in Jurkat cells by increasing the chromatin accessibility of HIV-1 (105). Moreover, the mechanisms of transcriptional repression acting on the integrated viral promoter are crucial for HIV latency regulation. Taube et al. conducted a CRISPR genome-wide knockout screen in Jurkat cells. They found that ZNF304 and TRIM28 could combine and be recruited to the viral promoter heterochromatin-inducing methyltransferases to silence HIV gene transcription (93).

Additionally, sufficient evidence suggests that metabolism changes in CD4^+^ T cells affect HIV infection (160–163), although the mechanism is poorly understood. In 2018, Xu et al. explored the host factors required for HIV latency using a genome-wide CRISPR knockout screen (164). They used an HIV-1 latent infection cell line and sorted cells for sequencing based on their GFP expression level (164). They found that knocking out *TSC1* or *DEPDC5* promoted HIV-1 reactivation in the T-cell line by influencing the mechanistic target of rapamycin complex 1 (mTORC1) pathway, a key driver of cell metabolism. Subsequently, in 2021, Deng et al. conducted a CRISPR knockout screen in an HIV-1 latently infected cell line (J-Lat 9.2) with a human CRISPR metabolic gene knockout library. They found that MAT2A-mediated one-carbon metabolism contributes to regulating HIV latency (102).

#### 5.1.4 Macrophages-mediated diseases

THP-1 is a human peripheral blood mononuclear cell line originally derived from a patient with acute monocytic leukemia. It is a suspension cell line and suitable for transfection or infection experiments. In 2023, Naderer et al. conducted a FACS-based genome-wide CRISPR/Cas9 screen in the THP-1 cell line to explore the mechanism between Panton–Valentine leukocidin (PVL) and complement C5a receptor 1 (C5aR1) (107). FBXO11 was identified as the potential target in macrophages for inflammation. Moreover, in 2019, a study performed a genome-scale CRISPR knockout library screen in THP-1 cells to identify potential genes involved in resistance to *Salmonella* uptake (94). *NHLRC2* was identified as the target gene involved in both *Salmonella* invasion and macrophage differentiation.

Additionally, the U-937 is another cell line that be used to study monocyte behavior and differentiation. It was originally isolated from a 37-year-old male patient with histiocytic lymphoma. In 2018, Superti-Furga et al. conducted a FACS-based CRISPR screen with a solute carrier (SLC)-knockout library in U-937 cells to specifically explore SLCs (108). SLCs participated in metabolic changes associated with phagocytosis of macrophages. They determined that SLC4A7 inhibits human intracellular microbicidal activity and limits the acidification of phagocytosed beads.

### 5.2 CRISPR screening in murine immune cells

#### 5.2.1 CD4^+^ T cells

##### 5.2.1.1 Regulatory T cells

Tregs are a small subset of immune cells that play a pivotal role in maintaining immune homeostasis. Dysregulation or dysfunction of Tregs can give rise to inflammatory disorders, including graft-versus-host disease (GvHD), transplant rejection, and autoimmune diseases (165). In 2022, Rathmell et al. designed a 1C metabolism gRNA library based on the Brie CRISPR knockout pooled library and conducted a CRISPR knockout screen in an ovalbumin (OVA)-induced lung inflammation model (128). They showed that MTHFD2 participates in proliferation and inflammatory cytokine production by regulating *de novo* purine synthesis and activation signals.

Moreover, one major barrier to immunotherapy in cancer is the immunosuppressive effect of the Tregs (128). Gaining insight into the mechanism governing Treg function would significantly contribute to identifying novel therapeutic targets for cancer treatment. Moreover, transcriptional regulator FOXP3 has been identified as a master regulator of Tregs and is activated throughout their development of the thymus (166). In 2020, Zheng et al. conducted a genome-wide knockout screen to explore the FOXP3 regulator in mouse primary Tregs using the Brie mouse CRISPR knockout pooled library. The cells were sorted for sequencing based on their Foxp3 expression level (111). The Brd9-containing non-canonical BAF complex was identified as a therapeutic target for manipulating Treg function by promoting Foxp3 expression.

Additionally, studies have increasingly found that nutrients interact with immunological signals to activate mTORC1, a key regulator of cell metabolism (167–170). Understanding the mechanism of nutrient signaling processes may be a novel insight into therapeutic target discover for immune-mediated disorders. In 2021, a study conducted a genome-wide CRISPR knockout screen in the Tregs using the Brie genome-scale sgRNA library. The cells were sorted for sequencing based on their phosphorylation of S6 (p-S6) level (113). Sec31a was found to interact with the GATOR2 component Sec13 to promote mTORC1 activation.

##### 5.2.1.2 Non-regulatory T cells

Recently, some studies used CRISPR screening to explore cell fate in non-regulatory T cells at the transcriptional and epigenetic levels, refining our understanding of CD4^+^ T cell biology. In 2021, Teichmann et al. conducted a genome-wide CRISPR screen in mouse naïve CD4^+^ T cells with the Teichmann retroviral mouse genome-wide CRISPR knockout library. The cells were sorted for sequencing based on different factors, including IL4 and IL13, XBP1, and GATA3 (120). They found that PPARG and BHLHE40 were the central networks regulating the differentiation of T helper 2 (Th2) cells. Additionally, the networks of lineage-specifying TFs controlling the specific differentiation of Th-cell subsets were affected by epigenetic processes (171). These epigenetic processes could change the genetic expression programs in Th cells (172). In 2023, a genome-wide CRISPR knockout screen was conducted in naive CD4^+^ T cells using a sgRNA library targeting 1,131 TFs to explore the differentiation mechanism of Th cells (109). The retrovirally transduced naïve CD4^+^ T cells of IL13-reporter mice (Rosa26^Cas9EGFP^ Il13^tdTomato^) were induced through the TCR and IL4 signaling pathway to shift their differentiation toward Th2 cells. The cells were sorted for sequencing based on their IL13^Tom^ expression (109). By acting as a crucial link between pioneer TFs and chromatin remodeling, the ADNP was found to be essential for immunological responses to allergens. Moreover, sufficient evidence suggests that metabolism is the guiding force in the differentiation of CD4^+^ T cells, which will undergo different metabolism changes throughout their life (173). One study used an *in vivo* screen to investigate the mechanisms underlying post-transcriptional and metabolic programs in T follicular helper (TFH) cell differentiation (124). Using a LCMV-Arm infection model, this study identified ETNK1, PCYT2, and SELENOI as specific post-transcriptional regulators selectively influencing TFH cell differentiation by enhancing CXCR5 surface expression and functional effects.

Additionally, CRISPR screening has also dramatically accelerated the therapeutic targets discovery in Th cells-mediated diseases in the last few years. Adoptive T-cell therapy (ATCT) contributes significantly to the therapeutic developments for cancer. Some studies have already found that coinjecting CD4^+^ and CD8^+^ T cells can improve and prolong antitumor activity (174, 175). While CD8^+^ T cells can clonally proliferate after antigen stimulation *in vivo*, intrinsic factors limit the proliferation of CD4^+^ T cells. In 2021, Menger et al. conducted a genome-wide CRISPR knockout screen in mouse CD4^+^ T cells. The transduced cells were sorted for sequencing based on their CFSE expression (121). They found that the SOCS1 acts as a nonredundant checkpoint inhibiting *in vivo* genome-wide CRISPR screens identify SOCS1 as intrinsic checkpoint of Th 1 cell proliferation. Moreover, systemic lupus erythematosus (SLE) is an autoimmune rheumatic disorder characterized by CD4^+^ T cell-mediated inflammatory responses that can result in tissue infiltration and organ damage (176). Recent studies have demonstrated a potential association between SLE pathogenesis and iron metabolism (177–179). In 2023, Rathmell et al. conducted a CRISPR knockout screen in naïve CD4^+^ T cells using a library targeting genes involved in iron metabolism (180). The transferrin receptor (TFRC/CD71) was identified as an activator of Th effector functions, including IL10 production, and an inhibitor of Tregs. Furthermore, the pathogenesis of helminth infections and allergic diseases is closely associated with Th2-mediated protective type 2 immune responses (181, 182) and Th2-dependent inflammatory responses (183–185). One study conducted a genome-wide CRISPR knockout screen in CD4^+^ T cells from IL13-reporter mice (Rosa26^Cas9^ Il13^tdTomato^) using the Brie mouse CRISPR knockout pooled library. The transduced cells were sorted for sequencing into brain finger protein BFP^+^ IL13^Tom+^ and BFP^+^ IL13^Tom−^ (110). They found that αvβ3-expressing Th2 cells could promote and enhance the Th2 response by forming multicellular factories such as T-T cell clustering and IL2/CD25/STAT5 signaling.

#### 5.2.2 CD8^+^ T cells

The studies based on CRISPR screens in mouse CD8^+^ T cells have mainly focused on the adoptive cell therapy (ACT). ACT can provide long-term benefits to cancer immunotherapy by isolating and engineering living T cells (186). The engineering of CD8^+^ T cells has sparked significant interest in ACT research due to its improved prognosis and specific cytotoxic capabilities (187–189).

The tumor model is mainly used in *in vivo* CRISPR screens to explore potential targets for ACT. Chen et al. conducted a genome-wide CRISPR knockout screen in OT1 cells using *Rag* ^−/−^ mice bearing E0771-mCh-OVA tumors, finding that DHX37 is a functional regulator of CD8^+^ T cells (119). In 2019, they also conducted an *in vivo* CRISPR screen to identify membrane targets for improving immunotherapy in GBM by transducing an adeno-associated virus (AAV)-surf library into naïve CD8^+^ T cells and transfusing them into GBM-bearing mice (126). In addition, another study transduced a lentiviral sgRNA metabolic sublibrary into activated OT1 cells and transfused them into B16-Ova melanoma-bearing mice to explore potential targets in ACT (112). They found that *Reg1*-deficiency promoted the persistence and effector function of the CD8^+^ T cells in tumors. Recently, Satpathy et al. also conducted a CRISPR knockout screen in the OT1 cell using a mini pool library, and the transduced cells were transfused into the *Rag* ^−/−^ mice six days after tumor inoculation (118). By sequencing the T cells from the tumors and spleen of the *Rag* ^−/−^ mice, they found that chromatin remodeling factors limit T cell persistence *in vivo*.

Moreover, the *in vivo* infectious model is another effective model in CRISPR screening research. In 2021, a study transduced a retroviral library into Cas9 CD8^+^ T cells, which have the P14-transgenic TCRs specific for LCMV gp3333–41, and then transfused the transduced cells into LCMV-infected mice (127). They found that genetic deletion of *Fli1* enhanced effector T cell (T_EFF_) differentiation in infections and cancer. Additionally, Chi et al. conducted a CRISPR knockout screen based on the *in vivo* LCMV infectious model to systematically investigate metabolic factors in T_EFF_ and memory T cell (T_MEM_) fate determination. The cells were sorted for sequencing based on their KLRG1 and CD127 expression (125). They demonstrated that amino acid transporters SLC7A1 and SLC38A2 inhibit T_MEM_ differentiation by modulating mTORC1 signaling. Furthermore, in 2022, Green et al. transduced naïve OT-I cells with a knockout library targeting epigenetic regulators and transfused them into the *Listeria monocytogenes*-OVA infected mice to explore T_MEM_ mechanisms (129). They identified the cBAF complex as a negative determinant of T_MEM_ cells.

While the above *in vivo* CRISPR screens allow for exploring cell mechanisms at a general level, they are highly susceptible to interference by various complex factors. Indeed, *in vitro* CRISPR screens are also an essential component in high-throughput cell biological research. In 2020, a study conducted a CRISPR knockout screen in CD8^+^ T cells using a gRNA library targeting 25 kinases known to maintain their activity in T cells for up to 16 hours after TCR stimulation (123). Cells were categorized into distinct phenotypes based on their expression of diverse molecular markers. The p38 Kinase was identified as a potential therapeutic target for adoptive immunotherapies. Moreover, Chen et al. conducted a comprehensive genome-wide CRISPR activation screen within a cytotoxicity assay (130). SIINFEKL peptide-incubated E0771 cells were co-cultured with transduced CD8^+^ T cells, with CD8^+^ CD107a^+^ T cells subsequently sorted for sequencing. They identified PRODH2 as a potential target for promoting CAR-T cell efficacy. LFA1 can interact with ICAM1 and ICAM2 to participate in T cell migration, adhesion, and activation (190). Schwartzberg et al. conducted a CRISPR knockout screen in CD8^+^ T cells using a PIP3-binding protein CRISPR library to identify genes influencing the binding ability of primary mouse T cells to ICAM1 (122). RASA3 was identified as the potential target gene by comparing sgRNA frequencies between scICAM1^neg^ (not binding ICAM1) and scICAM1^pos^ (ICAM1-binding) cells.

#### 5.2.3 B cells

Plasma cells are the primary source of antibodies, and the mechanism underlying their divergence from B cells has been thoroughly investigated (191–193). Disruptions in the typical antibody-secreting cell (ASC) formation pathway can cause immune-related diseases (132). The MAC-seq, which combines genetic analysis with quantitative methodologies, has been used to screen for drugs impacting the epigenetic machinery of B cells while assessing alterations in humoral immunity (132). Polycomb repressive complex 2 (PRC2) inhibitors were discovered that can support ASC differentiation in murine and human B cells *in vitro*. In addition, MYBL1, MYOF, GAS7, and ATOH8 were identified by functional dissection of downstream PRC2 effectors using an arrayed CRISPR screen (132).

Additionally, using a CRISPR/Cas9 screen, Wu et al. identified TCF3 and TCF4 and IRF4 as downstream effectors of p38 that govern plasma cell differentiation via *Blimp1* transcription (133). Moreover, in 2020, Laidlaw et al. identified HHEX as a TF controlling memory B cell differentiation using an inducible CRISPR/Cas9 screening strategy (134). Furthermore, aiming to better understand the IgE-BCR-mediated modulation of IgE responses, Newman and Tolar conducted a whole-genome CRISPR screen to compare the needs of IgE^+^ and IgG1^+^ B cells for plasma cell proliferation, survival, and differentiation (116). Interestingly, they found that the absence of calcium-calcineurin-NFAT pathway components improved IgE^+^ plasma cell development, whereas IgE^+^ plasma cells depended on the phosphoinositide 3-kinase PI3K-mTOR axis (116).

#### 5.2.4 Macrophages

Macrophages are a major component of the innate immune system and play a critical role in host-pathogen interactions (194). Microbial pathogens have evolved extensive defense systems to avoid being killed by macrophages (195). *L. monocytogenes* is a Gram-positive intracellular pathogen that can cause serious invasive infections (196). In 2023, Reniere et al. conducted an unbiased CRISPR/Cas9 screen using the Brie sgRNA library in immortalized bone marrow-derived macrophages (iBMDMs) to identify host factors important for *L. monocytogenes* infection (115). Tumor suppressor PTEN was found to promote macrophage phagocytosis of *L. monocytogenes*. Additionally, *Mycobacterium abscessus* was found to cause increasing community- and hospital-acquired infections in humans (197). In 2023, a study transduced murine macrophages with a genome-wide knockout library to generally define and characterize important regulators in macrophage-*M. abscessus* interactions, such as sulfated glycosaminoglycans(sGAG) (198). They found that macrophage-*M. abscessus* interactions were mainly regulated by sGAG. Moreover, *Histoplasma capsulatum* is a common pathogenic fungus that can live inside macrophage phagosomes (199) and causes the most common fungal respiratory disease in the United States (200). In 2022, Sil et al. performed a host-directed CRISPR/Cas9 screen in the mouse mononuclear macrophage cell J774A.1 cell line to explore the interaction mechanism between *H. capsulatum* and macrophages (135). They found that the C3a receptor (C3aR) was important in macrophages capturing pathogenic fungi.

We can also use CRISPR screening to explore the molecular mechanisms of different pathways involved in macrophage activation and function. IFN-γ promotes the proinflammatory responses of macrophages during infection (201). In 2020, Saeij et al. conducted a genome-wide CRISPR screen in bone-marrow-derived macrophages (BMDMs), discovering 353 toxoplasma genes determining parasite fitness in naїve or IFNγ-activated murine macrophages (136). They also found that parasites missing the dense granule protein GRA45 were less severe and more vulnerable to IFN-mediated growth suppression in mice. Moreover, in 2020, a study conducted a CRISPR screen in iBMDMs to explore genes essential for macrophage viability (137). The tumor necrosis factor (TNF) signaling cascade was found to have a suppressive effect on macrophages in an autocrine manner. Additionally, efferocytosis, which involves the clearance of apoptotic cells, is crucial for various processes, such as immune cell proliferation, tissue turnover, and organ regeneration (202). In 2022, Zhang et al. conducted a FACS-based genome-wide CRISPR knockout screen in primary mouse macrophages. They identified WDFY3 as a potential target for efferocytosis in macrophages (114). Futhermore, the N^6^-methyladenosine RNA modification can occur post-transcriptionally in almost all biological processes (203). However, its function in the innate immune cells is unknown. Li et al. identified major m6 A “writers” as the top candidate genes controlling macrophage activation in an RNA binding protein-focused CRISPR screen (131). They also discovered that *Mettl3* deficiency could inhibit TNF-α production in macrophages after lipopolysaccharide stimulation *in vitro*.

## 6 CRISPR screening in cancer immunology

Cancer is a genetic disease caused by cumulative genetic/epigenetic aberrations (204). An essential characteristic of cancer is immune evasion, primarily governed by checkpoints (205), which poses a substantial hurdle to cancer therapy. Fortunately, we can precisely identify the candidate genes whose mutation causes cancer with the help of the CRISPR screening, enabling us to explore appropriate drug targets and helping us understand cancer genomics (52). We summarized the current applications of CRISPR screens in the cancer cells and provided a summarization in **Table 7**.

**Table 7:** CRISPR screening studies in cancer immunology.

Name of library	Library Type	Library Size	Cell type	*In vivo* or *in vitro*	Screening methods	Reference
Mouse CRISPR Knockout Pooled Library (Brie)	Knockout	Genome-wide	Tumor cell	*In vivo*	Survival (under immune selective pressure)	(205)
Mouse CRISPR Knockout Pooled Library (Brie)	Knockout	Genome-wide	KPC cell	*In vitro*	Survival (under immune selective pressure)	(206)
Disease-related immune gene library (DrIM library)	Knockout	Custom	TNBC cell line 4T1	*In vivo*	Survival (under immune selective pressure)	(207)
Murine lentiviral CRISPR-Cas9 knockout (MusCK) library	Knockout	Genome-wide	TNBC cell line 4T1	*In vivo*	Survival (under immune selective pressure)	(208)
W. Nicholas Haining et al.	Knockout	Custom	B16 cell	*In vitro*	Survival (under immune selective pressure)	(209)
Epigenetic-focused sgRNA library	Knockout	Custom	KP cell	*In vivo* and *In vitro*	FACS (sorting of 15% PD-L1^hi^ populations and top 15% PD-L1^low^ populations for sequencing), Survival (under immune selective pressure)	(210)

Pancreatic ductal adenocarcinoma (PDA) is characterized by inherent immune cell deficiency and a distinctive TME composed of desmoplastic stroma (211) and suppressive immune cells (212). Frey et al. conducted *in vitro* and *in vivo* CRISPR screening to systematically explore PDA’s intrinsic mechanisms of immune evasion (206). They discovered that VPS4B and RNF31 are important in escaping CD8^+^ T cell killing. Additionally, immunotherapy is limited in patients with PDA due to inadequate T-cell infiltration and activation within the TME (213). Moreover, the CRISPR screens conducted by Fan et al. identified KDM3A as a potent epigenetic regulator of the immunotherapy response in patients with PDA (214). Furthermore, triple-negative breast cancer (TNBC) has the highest mortality and recurrence rates of all breast cancer types (215). Ji et al. conducted two-step customized *in vivo* CRISPR screens targeting disease-related immune genes (207). They identified LGALS2 as a potential immune escape regulator in different mouse models with multifaceted immune-deficiency characteristics.

Immune checkpoint blockade (ICB) is the primary immunotherapy in cancer care, and it has a considerable curative effect in many solid tumor types (216). Dubrot et al. conducted genome-scale CRISPR screens across cancer models treated with ICB, discovering that immune escape could be facilitated by inhibitory checkpoints involving IFN-mediated upregulation of classical and non-classical major histocompatibility complex (MHC) class I (205). However, the ICB is not appropriate for everyone with TNBC, and it can also cause additional side effects (217). Wang et al. conducted *in vivo* CRISPR screens, discovering that knockout of the *Cop1* in cancer cells decreased macrophage-associated chemokine secretion and tumor macrophage infiltration but enhanced antitumor immunity and ICB response in TNBC (208). An immune therapy blocking the PD1 checkpoint also had durable antitumor effects in different cancer types (218). However, it is only effective in a minority of patients (209). Manguso et al. conducted a CRISPR/Cas9 genome editing in transplantable mouse tumors treated with immunotherapy, confirming that defective IFN-γ signaling leads to immunotherapy resistance (209). Moreover, *TSC1* and *TSC2* are frequently mutated in non-small cell lung cancer (219). However, their functions in antitumor immunity remain unexplored. Huang et al. conducted *in vitro* and *in vivo* CRISPR screening in a murine *Kras* ^G12D^/*Trp53* ^−/−^ lung cancer model to systemically explore cell-intrinsic regulators of antitumor immunity (210). TSC1 and TSC2 were found to regulate PD-L1 expression *in vitro*. Additionally, they influenced the sensitivity to anti-PD1 treatment.

In conclusion, we can systematically explore genetic targets contributing to the immune escape mechanism using high-throughput CRISPR technology, which may provide new insights into the immune tolerance building of transplantation.

## 7 CRISPR technology in solid organ transplantation

### 7.1 Genomic modification of immune cells in organ recipients

Advancements in surgical techniques, high-quality perioperative care, efficient immunosuppressive agents, and effective post-transplant management of infectious diseases have substantially increased the success rate of organ transplantation. Therefore, organ transplantation has become the primary therapeutic approach for end-stage organ failure. However, the limited lifetime of the allograft is the primary barrier in organ transplantation. Twenty percent of recipients will lose their graft within five years, and 50% within 10–12 years (220). Allografts rejection, primarily regulated by the immune system, represents the central problem to the long-term survival of grafts. The interplay between the innate and adaptive immune systems, mediated by diverse immune cells, constitutes a key component of allografts rejection (221, 222). Furthermore, these immune cells can induce more vigorous immunological rejection in xenotransplantation. In recent years, numbers of studies have focused on the CRISPR genome editing of immune cells in recipients.

Firstly, Tregs have shown significant potential in ACT due to their feasibility, tolerability, and potential efficacy (165). Moreover, Tregs are pivotal target cells for establishing transplant tolerance (223). Until now, Treg therapy has consistently been shown to be a safe therapeutic method (224). It is important to minimize morbidity and mortality among solid organ transplant recipients and reduce the need for pan-immunosuppressive drugs (19). The first application of Treg therapy was in GvHD, and its therapeutic effect was demonstrated with data from preclinical models (165). In recent years, more and more studies used the CRISPR technology to modify the immune-modulating function of Tregs (225). In 2016, Levings et al. created an A2-CAR (HLA-A2–specific CAR) that markedly enhanced the therapeutic potential of Tregs in transplantation using CAR technology (225). Additionally, in 2017, Yoshimura et al. edited the *Foxp3* gene in mouse primary T cells using CRISPR/dCas9 (21). Moreover, in 2019, Horvat et al. used an activator-domain fusion-based dCas9 transcription activator to achieve prolonged *FOXP3* expression in Jurkat cells (22).

Besides Tregs, dendritic cells (DCs) are another potential gene-editing target for immune tolerance in organ transplantation. In 2019, Wang et al. used CRISPR/Cas9-based nanomedicine technology to directly cause CD40 deficiency in DCs *in vitro* and *in vivo* (226). They successfully induced a protective effect on graft survival by regulating T-cell activation.

In conclusion, the CRISPR genome editing technology might be a potential way to precisely and effectively intervene the immune cell function of organ recipients.

### 7.2 Genomic modification in organ donors

Another tricky issue for developing organ transplantation is the insufficient supply of donor organs. In 2020, the Organ Procurement and Transplantation Network reported that 116,577 patients were on waiting lists for organ transplantation in the USA (227). On average, 20 patients on the waiting list die every day because of the shortage of donor organs (228). Pre-implantation transcriptome modulation in allografts or xenografts have been proven a potential way to expand the donor pool in solid organ transplantation (229, 230).

In 2023, Keshavjee et al. used CRISPR/Cas technologies to upregulate IL10 expression in the donor lung and transplanted it into recipient rats (231). In addition, human and rat cell lines were tested for potency, titratability, and multiplexibility (231). This study has proved the feasibility of pre-implantation transcriptome modulation using CRISPR technology to expand the donor pool and improve transplantation outcomes. Moreover, using CRISPR gene editing, Schrepfer et al. removed the MHC I and II molecules and inserted CD47 into mouse and human induced pluripotent stem cells (iPSCs) to create hypoimmunogenic iPSCs that retain their pluripotent stem cell potential and differentiation capacity (232). In totally MHC mismatched allogeneic recipients, endothelial cells, smooth muscle cells, and cardiomyocytes created from hypoimmunogenic mouse or human iPSCs reliably evaded immune rejection and survived over the long term without needing treatment (232). These findings suggest the possibility of creating hypoimmunogenic cell grafts for future universal transplantation.

In the xenotransplantation field, increasing evidence suggests that pigs may be the best xenograft source (230) because of their size, availability, breeding characteristics, and physiological similarities to humans (228). However, the major problem with xenotransplantation is the immune barriers between pigs and humans (233, 234). In recent years, advances in the genetic modification of pigs with CRISPR-Cas9 have contributed significantly to the advancement of xenotransplantation (233). In 2014, Zhou et al. created the first CRISPR/Cas9-engineered pig and developed an efficient one-step method to generate genome-modified pigs by zygote injection of the CRISPR/Cas system (235). This method suggests the potential of CRISPR technology to swiftly establish substantial animal models for human research. Moreover, subsequent studies have used CRISPR technology to knockout glycosyltransferase and other genes (*PERV*, *GHR*, *ULBP1*, and *SLA-1*) or knock-in one gene (an anti-CD2 monoclonal antibody transgene into *GGTA1*) in pigs (236). Overall, there is ample evidence to demonstrate that CRISPR technology has emerged as an effective tool in xenotransplantation research.

## 8 Prospective applications of CRISPR technology in solid organ transplantation

As mentioned above, rejection caused by immune cells and the shortage of donor organs are the two significant barriers to developing organ transplantation. Impeding the function of immune cells in rejection and genetically modifying the graft to escape the immune system are the major tasks in organ transplantation. Additionally, with the rapid development of CRISPR technology, we can make precise and stable genome edits in immune cells and grafts. However, current studies have identified few genetic targets that can effectively build immune tolerance in transplantation. Advancements in high-throughput CRISPR technology have accelerated the discovery of potential therapeutic targets in immune cells and cancer cells. This provides a novel insight into identifying therapeutic targets in transplantation. Genome-wide screening of the immune or non-immune cells and intervening these targets using the CRISPR/Cas9 system may be a novel horizon in transplantation research.

### 8.1 Conducting CRISPR screening in solid organ transplantation

#### 8.1.1 CRISPR screening in immunocytes of organ recipients

Immunocyte-mediated rejection is the primary barrier to building long-term graft survival. There is an urgent need to explore effective approaches to modulate immunocytes in transplantation. However, few treatments are available for immunocyte intervention despite significant advances in transplantation in recent decades. Most immunosuppressive agents have successfully improved short-term graft survival, but long-term graft survival has stagnated. Fortunately, advancements in CRISPR/Cas9 genome editing technology make it possible to conduct precision medicine based on immunocytes in transplantation. In the last decade, CRISPR screening has been widely used in different immunological diseases to explore many potential targets in immunocytes, providing novel insights into transplantation.

##### 8.1.1.1 CRISPR screening in alloreactive T cells

As mentioned above, CRISPR screening has been widely applied to T cells with a particular focus on improving immune function in cancer (237), suppressing the inflammatory effect in autoimmune disease (11), and enhancing antiviral function in infectious diseases (238). Sufficient evidence demonstrates that CRISPR screening is an effective method for detecting potential targets in T cells. Based on the clinical experience, the therapeutic agents that can modulate the function of alloreactive T cells have the considerable effects on long-term graft survival. By understanding how alloreactive T lymphocytes recognize donor antigens, we can intervene them more effectively in solid organ transplantation (239). Conducting CRISPR screens in alloreactive T cells may be a novel approach to exploring potential targets for long-term graft survival.

The current applications of CRISPR screening in T cells have mainly focused on Tregs, Th cells, and cytotoxic T cells. These cells also play an important role in transplant rejection and can be the suitable cell models for CRISPR screening in transplantation. With advances in CRISPR screening, we can use multiple approaches to explore the genetic mechanism underlying different effector cell phenotypes in transplantation, including cell fate, effector function, and cell exhaustion.

For *in vitro* CRISPR screening, we can use a suitable gRNA library to target alloreactive T cells and apply different intervention factors to the transduced cells. Then, the different alloreactive T cells phenotypes can be selected by FACS or MACS based on the multiple cell markers. After sequencing, the genetic mechanism underlying different phenotypes can be elaborated. For *in vivo* CRISPR screening, the alloreactive T cells can be transduced with the gRNA library *in vitro.* Then, the transduced cells can be transferred into various animal transplantation models. The different intervention approaches can also be conducted in animal models. Finally, the cells for sequencing can be sorted based on different cell makers, and the target genes can be validated using the sequencing results.

##### 8.1.1.2 CRISPR screening in alloantigen APCs

By directly interacting with T cells, APCs orchestrate distinct functional outcomes of the immune response (240). Therefore, exploring effective approaches to modulate APCs is one major task for transplantation. In transplantation, dying graft cells release inflammatory molecules that trigger APC maturation and T-cell response (241). As mentioned above, one study successfully used the CRISPR/Cas9-based nanomedicine technology to directly cause CD40 deficiency in DCs to create a protective effect on graft survival. This study showed that CRISPR-based genomic editing may be an effective method to make precise genomic edits in APCs. Moreover, using CRISPR screening, we can explore the potential targets at the genome-wide scale. The CRISPR screens conducted in APCs have mainly focused on the host-pathogen interaction of macrophages. Macrophages have been shown to be a reliable and stable cell model for CRISPR screening in APCs. In transplantation, after antigen stimulation (*in vitro* or *in vivo*), the transduced cells can be sorted for sequencing using different cell markers, such as the MHC. Then, the candidate genes are validated using the sequencing results. Finally, multiple antigen presentation assays can be used to validate the candidate genes in APCs.

#### 8.1.2 CRISPR screening in organ donors

CRISPR screens in non-immune cells have mainly focused on the immune escape mechanism of cancer cells under immune pressure. In organ transplantation, allograft rejection-mediated immune pressure is also an important factor affecting graft survival, significantly shrinking the donor pool for organ transplantation. Performing CRISPR screens in the grafts will accelerate the discovery of resistance target genes to immune pressure. Additionally, combining donor organ *ex vivo* perfusion systems and CRISPR gene editing technology increases the time window and enhances the capability for pre-implantation transcriptome editing of grafts. Identifying resistance target genes using CRISPR screens and then using CRISPR technology to modify grafts may be a novel approach for transplantation.

For *in vitro* CRISPR screens in grafts, the transduced cell can be applied with a killing assay mediated by the cytotoxic cells in allograft rejection. Then, the surviving cells can be sorted for sequencing to explore the resistance genes for allograft rejection. Additionally, Pu et al. successfully conducted an *in vivo* CRISPR screening in Cas9 mutagenesis mice using an AAV gRNA library to explore potential targets for cardiomyocyte maturation (242). This approach may be a reliable tool for conducting *in vivo* CRISPR screens in grafts. After transducing the grafts with the AAV gRNA library, they could be transplanted into an allogeneic animal model to create immune pressure *in vivo*. Then, the graft cells could be sorted for sequencing to identify the potential therapeutic targets. Additionally, various biomedical methods can be used to validate the therapeutic effects of the candidate genes.

### 8.2 The overview of CRISPR technology in solid organ transplantation

**Figure 5:**
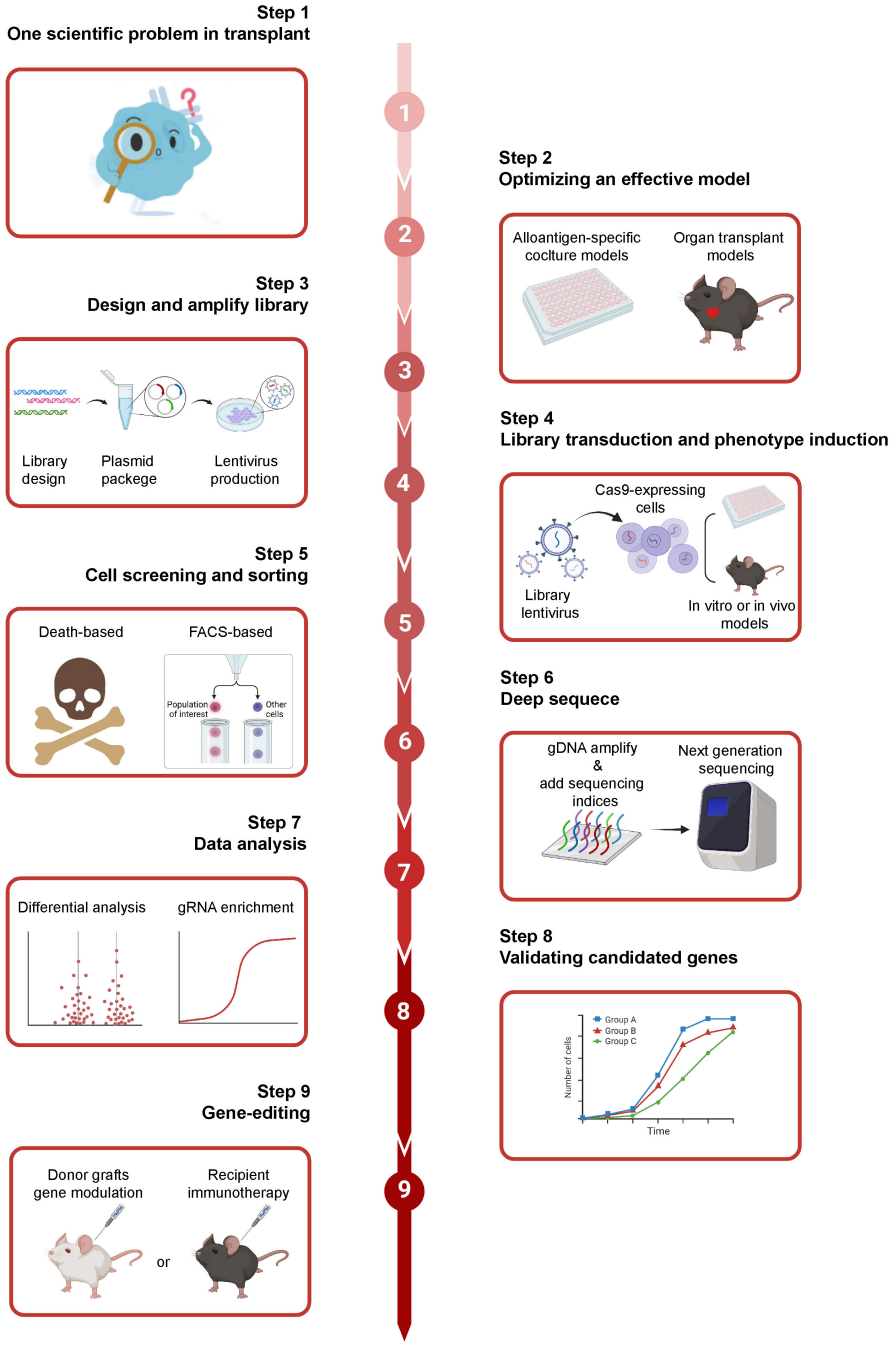
The overview of CRISPR screening in solid organ transplantation. Firstly, we should have a scientific question. Subsequently, an appropriate model can be chosen based on the scientific question. And, an optimized gRNA library (custom or genome-wide) should be designed, followed by pooled sgRNA synthesis, plasmid cloning, and viral particle production. Additionally, after transducing the gRNA library into the target cells, they can be sorted for sequencing based on positive or negative selection or different phenotypes. The genomic DNA (gDNA), the global genetic information of sorted cells, need to be isolated and amplified for further analysis. Finally, the potential targets in the donor grafts and immunotherapy can be intervened with the CRISPR/Cas 9 based genome editing, giving the novel insights for clinical problems. Created with BioRender.com.

By combining CRISPR screening with traditional CRISPR technology in transplantation research, we conceive an overview specific to solid organ transplantation studies. The main protocol steps are provided in **Figure 5**. First, we should have a scientific question. Second, an appropriate model should be chosen based on this question. Third, an optimized gRNA library (custom or genome-wide) should be designed, followed by pooled sgRNA synthesis, plasmid cloning, and viral particle production. Fourth, after transducing the gRNA library into the target cells, they should be sorted for sequencing based on positive or negative selection or different phenotypes. Fifth, the genomic DNA (gDNA), which contains the global genetic information of sorted cells, need be isolated and amplified for further analysis. Sixth, various methods should be used to validate candidate genes identified from the sequencing results. A mini-pool gRNA library can be designed based on these candidate genes to enable their more precise targeting. Finally, after identifying candidate genes, we can genetically modify grafts or immune cells to build immune tolerance in clinical applications or animal models.

### 8.3 The challenges of CRISPR technology in solid organ transplantation

Firstly, the delivery of Cas9 to certain cell types is often constrained by low transfection and transduction efficiency, particularly in primary immune cells and *in vivo* (243). Up to now, electroporation may be a potential way to solve this. Additionally, in CRISPR knockout screenings, certain perturbed cells might retain target protein function due to in-frame editing (244). And, verifying individual cell edits is usually not possible which increases the screen’s noise signals. Off-target effects are also a major potential problem for CRISPR genome editing (245). Incorrect gRNA binding will lead to undesired off-target mutations, potentially leading to genome instability and disrupting the normal functions of genes. CRISPR/Cas9 genome editing can also lead to massive base loss, which is potentially a more severe outcome than off-target effects (246). Furthermore, on-target mutagenesis in double-strand breaks which are caused by single-guided RNA/Cas9 can produce the long-range transcriptional consequences and pathogenic consequences (247). Finding the efficient tools that can make more precise genome edits is urgent in the field of CRISPR genome editing.

Moreover, the immune response is another severe issue in CRISPR genome editing. The gRNAs and DNA plasmids can activate the pattern recognition receptors that sense the foreign nucleic acids. For example, a triphosphate group at the 5’ end of gRNAs in the ribonucleoprotein (RNP) complex can be recognized as a foreign viral RNA by the RIG-I receptor, inducing the type I IFN-mediated immune response that mediates a cytotoxic effect (248, 249). Moreover, adenoviral vectors can also induce an inflammatory response, impeding the development of *in vivo* genome intervention (250–252). Thus, safe and efficient delivery systems is urgent in therapeutic genome editing in transplantation.

## Conclusion

In recent years, CRISPR/Cas9 has proven a promising strategy for precisely altering multiple genetic targets in previously untreatable disorders. It has already shown potential therapeutic value in solid organ transplantation, such as CAR-T therapy for Allograft rejection and genetic modification in xenotransplantation. Especially, combining CRISPR/Cas9-based gene editing with an *ex vivo* organ perfusion system would enable pre-implantation transcriptome editing of grafts. Additionally, a high-throughput CRISPR screening technology has shown a powerful ability to determine the genetic mechanisms behind specific phenotypes in the genomic level. Genome-wide screening of the immune or non-immune cells and intervening these targets using the CRISPR/Cas9 system may be a novel horizon in transplantation research.

## Author contributions

XL: Writing – original draft. ZC: Writing – original draft. WY: Writing – original draft. JY: Writing – review & editing. XZ: Writing – review & editing. YL: Writing – review & editing. YN: Writing – review & editing. SR: Writing – review & editing. SW:Writing – review & editing. ZL: Writing – review & editing. JLZ: Writing – review & editing. YH: Writing – review & editing. JJZ: Writing – review & editing. CX: Writing – review & editing. JX: Writing – review & editing. JW: Writing – review & editing.

## Glossary

**Table:**

RNAi	RNA interference
pre-crRNA	precursor CRISPR RNA
sgRNA	single-stranded guide RNA
tracrRNA	trans-activating crRNA
dCas9	dead Cas9
nCas9	Cas9 nickase
HNH	histidine/asparagine
DSBs	DNA double-strand breaks
pegRNA	prime-editing guide RNA
shRNA	short hairpin RNA
PCR	polymerase chain reaction
FACS	fluorescence-activated cell sorting
MACS	magnetic-activated cell sorting
PAM	protospacer-adjacent motif
QC	quality control
CAR	Chimeric antigen receptor
TME	tumor microenvironment
GBM	glioblastoma multiform
ART	Antiretroviral therapy
HIV	human immunodeficiency virus
GFP	green fluorescent protein
IFN-Is	Type I interferons
ISGs	IFN-stimulated genes
CRISPRi	CRISPR interference
mTORC1	rapamycin complex 1
Treg	regulatory T cell
TF	transcription factor
TCR	T cell receptor
FASL	Fas ligand
PVL	Panton–Valentine leucocidin
C5aR1	C5a receptor 1
SLC	solute carrier
Th2	T helper 2
TFH	T follicular helper
GvHD	graft-versus-host disease
OVA	ovalbumin
ATCT	adoptive T-cell therapy
SLE	systemic lupus erythematosus
ACT	Adoptive cell therapy
AAV	adeno-associated virus
T_EFF_	effector T cell
T_MEM_	memory T cell
ASC	antibody-secreting cell
PRC2	Polycomb repressive complex 2
iBMDMs	immortalized bone marrow-derived macrophages
sGAG	sulfated glycosaminoglycans
C3aR	C3a receptor
BMDMs	bone-marrow-derived macrophages
TNF	tumor necrosis factor
PDA	Pancreatic ductal adenocarcinoma
TNBC	triple-negative breast cancer
ICB	Immune checkpoint blockade
MHC	major histocompatibility complex
APCs	antigen-presenting cells
DCs	dendritic cells
iPSCs	induced pluripotent stem cells
RNP	ribonucleoprotein
gDNAs	genomic DNA
